# Manure properties, soil conditions and managerial factors regulate greenhouse vegetable yield with organic fertilizer application across China

**DOI:** 10.3389/fpls.2022.1009631

**Published:** 2022-10-21

**Authors:** Yangzhou Xiang, Yuan Li, Xuqiang Luo, Ying Liu, Xuejiao Yue, Bin Yao, Jianming Xue, Leiyi Zhang, Jing Fan, Xiuyue Xu, Yonghua Li

**Affiliations:** ^1^ Guizhou Provincial Key Laboratory of Geographic State Monitoring of Watershed, School of Geography and Resources, Guizhou Education University, Guiyang, China; ^2^ The State Key Laboratory of Herbage Improvement and Grassland Agro-ecosystems of Lanzhou University, National Field Scientific Observation and Research Station of Grassland Agro-Ecosystems in Gansu Qingyang, College of Pastoral Agriculture Science and Technology, Lanzhou, China; ^3^ School of Biological Sciences, Guizhou Education University, Guiyang, China; ^4^ Institute of Desertification Studies, Chinese Academy of Forestry, Beijing, China; ^5^ Institute of Ecological Conservation and Restoration, Chinese Academy of Forestry, Beijing, China; ^6^ College of Biology and the Environment, Nanjing Forestry University, Nanjing, China; ^7^ New Zealand Forest Research Institute Ltd (Scion), Scion, New Zealand; ^8^ South China Institute of Environmental Sciences, Ministry of Ecology and Environment of the People 's Republic of China (PRC), Guangzhou, China

**Keywords:** meta-analysis, organic fertilizer, plastic shed production systems, vegetable yield, soil properties

## Abstract

To better understand the responses of vegetable yields in a greenhouse system to organic fertilizer through a quantitative evaluation based on peer-reviewed journal articles and in consideration of environmental managerial factors. We conducted a meta-analysis of 453 paired observations from 68 peer-reviewed journal articles to assess the response of vegetable yields in greenhouse vegetable systems in China to organic fertilization. Compared with the control (no organic fertilizer), organic fertilization significantly increased the yields of vegetables by 44.11% on average. The response of vegetable yields to organic fertilizer tended to increase with the increasing experimental duration. Organic fertilizer application had the greatest potential for leafy vegetables (+76.44%), in loamy soils (+53.94%), at moderate organic fertilizer carbon input levels (+54.13%), and in soils with moderate initial soil total nitrogen levels (+50.89%). Aggregated boosted tree analysis indicated that organic fertilizer carbon inputs, vegetable type and experimental duration were the predominant factors that manipulated the response of vegetable yields to organic fertilizer application. The rational application of farmyard manure would be a promising strategy for increasing vegetable yields in greenhouse vegetable systems in China. Factoring in vegetable type, carbon and nitrogen inputs of organic fertilizer, and soil texture would benefit vegetable yields with the application of organic fertilizer.

## Introduction

Vegetables provide various compounds to human diets, including dietary fiber and several minerals, vitamins, phytochemicals, and secondary metabolites, and they confer beneficial biological effects and prevent certain noncommunicable diseases with regular consumption (Yahia et al., 2019). China has the largest vegetable cultivation area and produces the most vegetables in the world (Wang et al., 2019b). From 1999 to 2018, the cultivation area and the production quantity of the vegetable system in China increased by 132% and 208%, respectively (FAO, 2020).

Compared with open-field cultivation, protected vegetable production, typically results in greenhouse vegetable systems that can grow a larger variety of vegetables (Li et al., 2019a), especially during the colder seasons or in high-altitude areas. Greenhouse vegetable systems are widely practised in China and account for 55% of the total vegetable cultivation area (Liang et al., 2019). However, the excessive application of synthetic nitrogen (N) fertilizers *via* conventional flooding irrigation is often utilized in greenhouse vegetable systems in China has led to a series of environmental issues, such as nutrient leaching (Xu et al., 2020), soil degradation (Bai et al., 2020), and a reduction in the yield and quality of vegetables.

Farmers and researchers have adopted additional strategies to overcome those issues in the vegetable production system, such as vegetable rotation (Li et al., 2009), summer fallow periods (Tian et al., 2010), intercropping (Ding et al., 2020), soil solarization (Ichihashi et al., 2020), and the application of organic fertilizer (Zhou et al., 2019a). In particular, incorporating manure with vegetable production would be a promising approach to appropriately utilize livestock wastes and environmentally friendly enhance vegetable production (Cai et al., 2019; Zhen et al., 2020), because the largest livestock production in the world occurs in China (Bai et al., 2016), and about 4.6 billion tons of manure are generated each year (Du et al., 2020). Increased livestock wastes badly impact water and air quality through greenhouse gas emissions and nutrient leaching (Bai et al., 2016).

Previous studies have shown that the application of organic fertilizer was an eco-friendly strategy for sustainable vegetable production (Yi et al., 2021), this practice can enhance vegetable yields (Zhou et al., 2019a), reduce nutrient losses (Xu et al., 2020), decrease greenhouse gas emissions (Gruda et al., 2019), control vegetable diseases (van Bruggen et al., 2015), and maintain soil health (Luan et al., 2019). However, previous studies have reported inconsistent results regarding organic fertilizer addition in greenhouse vegetable systems. Some studies have shown increases in vegetable yields (Zhang et al., 2019; Zhou et al., 2019a). In contrast, others have shown significant decreases in vegetable yield (Cesarano et al., 2017; Xu et al., 2020). Some have shown no changes in response to organic fertilizer application (Wang et al., 2017). These conflicting results may be owing to factors, such as vegetable type, cultivation history, fertilization regime, organic fertilizer type, fertilizer amounts and soil properties (Cesarano et al., 2017; Wang et al., 2017). Thus, the effects of organic fertilizer application on vegetable production in greenhouse vegetable systems are still uncertain.

To better understand the responses of vegetable yield in greenhouse vegetable systems to organic fertilizer, it is crucial to perform a quantitative evaluation based on peer-reviewed journal articles and take environmental factors (e.g., soil nutrient status) and managerial factors (e.g., vegetable type, organic fertilizer type, and fertilizer amounts) into account. A meta-analysis can quantify changes in vegetable yield in greenhouse vegetable systems with organic fertilizer and determine the driving factors responsible for the yield discrepancy in the greenhouse vegetable system using organic fertilizers (Liu et al., 2021).

Here, the meta-analysis, based on the plot experiments of vegetable yield in greenhouse vegetable systems in China, aimed to (i) investigate the overall effects of the application of organic fertilizers on vegetable yield; (iii) examine which factors dominate the responses of vegetable yield to the application of organic fertilizers; and to test the hypothesis that effects of organic fertilizers on vegetable yields would vary with manure properties (e.g., fertilizer type, the proportion of organic N, inputs of organic fertilizer C and N), soil properties (e.g., initial soil pH, initial soil organic carbon (SOC) or total N (TN) content, and soil texture), and managerial factors (e.g., vegetable types and experimental duration).

## Materials and methods

### Literature search

We identified peer-reviewed publications published between 1990 and April 2022 that investigated the effects of organic fertilizers on greenhouse vegetable systems in China using the China National Knowledge Infrastructure (http://www.cnki.net/) and ISI-Web of Science (http://apps.webofknowledge.com/) with the search terms “organic fertilizer” or compost or manure or straw or “green manure” or “commercial organic fertilizer” and “greenhouse vegetable” or “tunnel vegetable” or “intensive cultivation” or “vegetable under plastic shed production systems and yield or production”.

### Data collection criteria

The following criteria were used for study selection: (1) the studies reported data collected from plot trials under greenhouse vegetable systems, excluding pot or open-field experiments; (2) the studies were conducted in China; (3) the studies included treatment with only organic fertilizer or organic fertilizer in addition to inorganic fertilizer (base fertilizer) and control (without fertilizer); (4) the studies specified the content of N in the organic fertilizers; (5) the studies reported vegetable yield with means, standard deviations (SD) or standard errors (SE), and had at least three replicates; (6) if the study included different vegetable species, we considered them distinct observations; and (7) if the study included several experiments under different abiotic conditions, such as experimental locations, fertilization regimes or organic fertilizer types, we considered them as separate studies. Finally, we compiled 453 pairs of data comparing the vegetable yield between the treatment group (organic fertilizer) and the control group (no organic fertilizer) from 68 references for meta-analysis (Online Resources **Table S1** and **Notes S1**).

### Data extraction

We collated the data from each study on the vegetable yield, location (e.g., longitude and latitude), vegetable species, experimental duration, fertilization regimes, organic fertilizer feedstocks, organic fertilizer nutrients (e.g., C and N inputs), synthetic N inputs, and initial soil properties (e.g., soil texture, pH, soil organic matter [SOM], SOC, and TN). Most of the data were either obtained from tables and text or extracted from figures using GetData (version 2.26) Graph Digitizer software (http://getdata-graph-digitizer.com/download.php). Additionally, we calculated certain indices, such as SOC=SOM/1.724 (Zhou et al., 2019b), and TN inputs = organic fertilizer N inputs + synthetic N inputs. The equation of pH[H_2_O]=1.65 + 0.86 × pH[CaCl_2_] was used to convert the soil pH 1:5 CaCl_2_ to 1:5 H_2_O (Minasny et al., 2011; Li et al., 2019b). Soil texture datasets were extracted from the International Institute for Applied Systems Analysis (Fischer et al., 2008) for the longitude and latitude of the experimental sites when information was not available in peer-reviewed publications.

The vegetables cultivated were classified into three groups based on their edible parts (Dong et al., 2020): (1) fruit vegetables, including tomato (*Solanum lycopersicum*), cucumber (*Cucumis sativus*), pepper (*Capsicum annuum*), eggplant (*S. melongena*), sweet pepper (*Capsicum frutescens*) and cherry tomato (*S. lycopersicum* var. *cerasiforma*); (2) leafy vegetables, including purple cabbage (*Brassica oleracea* var. *capitata f. ruba*), pak choi (*B. napus* subsp. *chinensis*), spinach (*Spinacea oleracea*), head cabbage (*B. oleracea*), cabbage (*B. oleracea* var. *capitata*), fennel (*Foeniculum vulgare*), lettuce (*Lactuca sativa*), amaranth (*Amaranthus* spp.), baby bok choy (*B. napus* subsp. *chinensis*), coriander leaf (*Coriandrum sativum*), tung choy (*Glebionus coranaria*), chrysanthemum (*Chrysanthemum* spp.) and rapeseed (*B. napus*); and (3) stem vegetables, such as celery (*Apium graveolens*). The experimental durations were classified into categories of<3, 3-10 and >10 years, representing short-, medium- and long-term experiments, respectively (Zhang et al., 2020). The amendment type datasets were divided into two categories as described by Masunga et al. (2016): (1) non-composted organic fertilizer, which included raw slurry and farmyard manure, and (2) composted organic fertilizer, which included farmyard manure after composting or anaerobic digestion. The values of the proportion of N applied *via* organic fertilizer to the total N inputs (particulate organic nitrogen [Pon]) were classified into four categories: 0<Pon ≤ 25%, 25%<Pon ≤ 50%, 50%<Pon ≤ 75% and 75%<Pon ≤ 100% (Xia et al., 2017). The C inputs from organic fertilizers (organic fertilizer C inputs) were classified into three categories:<5,000, 5,000-10,000 and >10,000 kg C ha^-1^yr^-1^. As in a previous study (Wang et al., 2018), the N inputs from organic fertilizers (organic fertilizer N inputs) were classified into three categories:< 200, 200-400 and >400 kg N ha^-1^ yr^-1^. The initial soil pH values were classified into three categories:<6.5, 6.5-7.5 and >7.5 as described by Xiao et al. (2019). Based on the distribution of the data-sets in our study, the initial soil organic carbon content (initial SOC) values were classified into three groups following Du et al. (2020):<12, 12-20 and >20 g kg^-1^; the initial soil TN was divided into three groups as described by Zhang et al. (2020):<1, 1-2 and >2 g kg^-1^. In accordance with (Dlamini et al., 2016), the soil texture in each study was categorized as sand (< 20% clay), loam (20-32% clay) and clay (> 32% clay).

### Data analysis

The natural log-transformed response ratio (ln*RR*) was employed to quantify the effect of organic fertilizer on vegetable yield following Hedges et al. (1999) using Eq. (1):


(1) $$lnRR=ln\left(x_{t}/x_{c}\right)$$

where ln*RR* is the ratio of the mean value of the vegetable yield in the amendment treatment group, *x*_t_, to that in the control group, *x*_c_.

The weighting factor (*w_i_*) for each study was calculated following Eq. (2):


(2) $$w_{i}=\left(n_{t}\times n_{c}\right)/\left(n_{t}+n_{c}\right)$$

where *w_i_* is the weight associated with each ln*RR* observation, and *n*_t_ and *n*_c_ are the number of replications in the organic fertilizer treatment and control groups, respectively.

The weighted effect sizes for each ln*RR* of the organic fertilizer treatment and control groups were calculated using Eq. (3):


(3) $$lnRR_{++}=\sum \left(lnRR_{i}*w_{i}\right)/\sum w_{i}$$

where ln*RR_i_* is the effect size (ln*RR*) for the *i^th^* study, and *w_i_* is the weight for the *i^th^* study.

The weighted standard error (SE) was calculated using Eq. (4):


(4) $$S(\ln RR_{++})=\sqrt{1/\sum w_{i}}$$

The 95% confidence interval (95% CI) was calculated using Eq. (5):


(5) $$95\%\;CI=\;lnRR_{++}\pm \;1.96S\left(lnRR_{++}\right)$$

The means and bias-corrected 95% CIs of the estimated effect size for each observation were generated using a bootstrapping procedure with 4,999 iterations (Shakoor et al., 2021). Only groups with more than two valid comparisons were included in our meta-analysis. If the 95% CI did not include zero, the effect of organic fertilizer was significant. To facilitate interpretation, the percentage changes in vegetable yields were computed following Eq. (6): [*exp* (*lnRR*_++_)−1] × 100*%* . The frequency distribution of the effect sizes was fit to a Gaussian distribution function for vegetable yields to test the homogeneity of observations (Gurevitch and Hedges, 1999; Xiang et al., 2021). A chi-square test determined whether the heterogeneity *Q*_total_ among the ln*RR* of changes in vegetable yield with organic fertilizer treatment significantly exceeded the expected sampling error (Hedges et al., 1999). The fail-safe number was used to test for publication bias; the results of a meta-analysis can be considered robust and reliable if this number is higher than 5×*n* + 10 (where *n* is the number of observations included in the study) (Rosenthal, 1979).

Ordinary least squares (OLS) regression analysis was used to analyze the relationships of the vegetable yield response ratios to the experimental duration, Pon, organic fertilizer C inputs, organic fertilizer N inputs, initial soil pH, initial SOC, initial soil TN, and soil texture. Aggregated boosted tree (ABT) analysis was conducted using the “gbmplus” package in R software version 3.6.3 (Wang et al., 2021) with 500 trees for the boosting, a 0.02-fold shrinkage rate and three-way interactions. This analysis was used to quantitatively and visually assess the relative effects of the experimental conditions (experimental duration and vegetable type), organic fertilizer properties (organic fertilizer type, Pon, organic fertilizer N inputs, and organic fertilizer C inputs) and edaphic factors (initial soil pH, initial SOC, initial soil TN, and soil texture) on the yields of vegetables.

All statistical analyses and plots were carried out in MetaWIN 2.1 and SigmaPlot 12.5 (Systat Software, Inc., San Jose, CA, USA), respectively.

## Results

### Overview of the dataset

**Figure 1:**
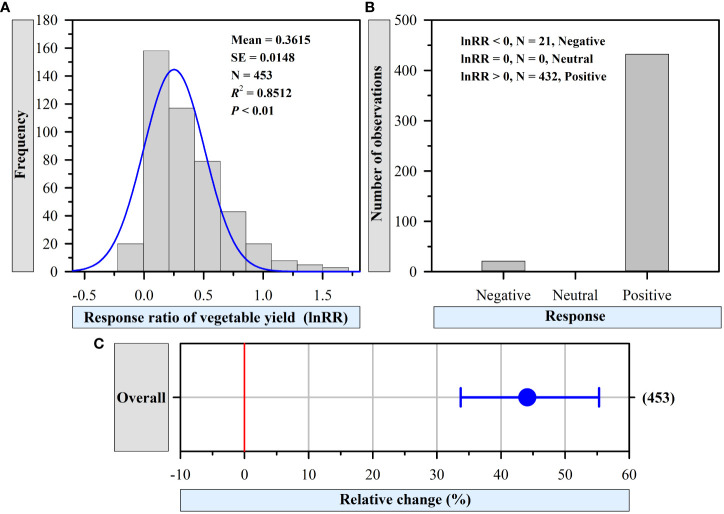
Frequency distribution of response ratio (lnRR) **(A)**, numbers of response **(B)**, and overall effect **(C)** of vegetable yield with organic fertilizer compared to that with no fertilizer. The blue fitted curve is the estimated Gaussian distribution of the frequency, and N represents the number of comparisons. The number of observations is shown right parenthesis. The blue horizontal bar represents the 95% confidence interval (CI). The CI does not overlap with 0 red line, which indicates significant positive effect at *P* < 0.05.

A total of 453 paired observations from 68 peer-reviewed journal articles were included in the database. All the studies included were conducted in China. Selected articles were published between 2003 and 2020, and 79% were published in the past decade. The Shapiro-Wilk test of the Gaussian distribution fitting (*p* > 0.05) indicated that the datasets were homogenous (**Figure 1**).

The fail-safe numbers for the database of vegetable yields by vegetable type (14,781.9), experimental duration (14,781.9), organic fertilizer type (14,781.9), Pon (14,781.9), organic fertilizer C inputs (7,126.5), organic fertilizer N inputs (14,781.9), initial soil pH (11,044.8), initial SOC (14,611.4), initial soil TN (8,141.4) and soil texture (14,687.8) were significantly higher than the 5n+10 threshold (where n is the number of observations) over which the mean effect size can be considered robust.

### Effect of organic fertilizer on vegetable yield depended on the vegetable type

**Figure 2:**
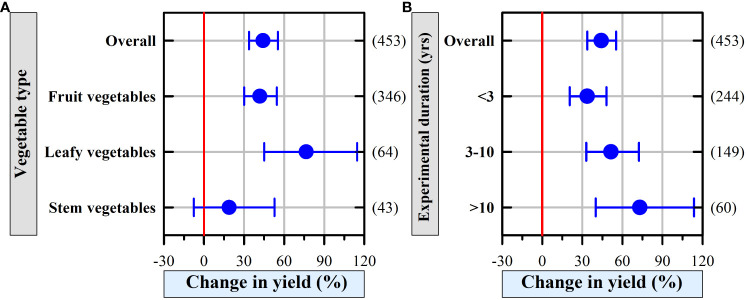
The effects of managerial factors **(A)** vegetable type and **(B)** experimental duration on greenhouse vegetable yield. The numbers of observations are shown right parentheses. The blue horizontal bars represent the 95% confidence intervals (CIs). If the CI does not overlap with 0 red line, which indicates a significant effect at *P* < 0.05.

Across all observations, the application of organic fertilizer significantly increased the vegetable yield by 44.11% except for stem vegetables (**Figure 2A**; mean: 18.82%, CI: -7.59 to 52.76%). The yields of fruit vegetables and leafy vegetables significantly increased by 47.15% (CI: 30.06% to 54.50%) and 76.44% (CI: 45.05% to 114.64%), respectively.

Organic fertilizer addition significantly increased vegetable yield regardless of experimental duration (**Figure 2B**), and the vegetable yield responded more positively to the duration of organic fertilizer addition. However, >10 years of experiments had increased variances (CI: 40.07% to 113.57%; **Figure 2B**).

### Organic fertilizer increased vegetable yield irrespective of fertilizer properties

**Figure 3:**
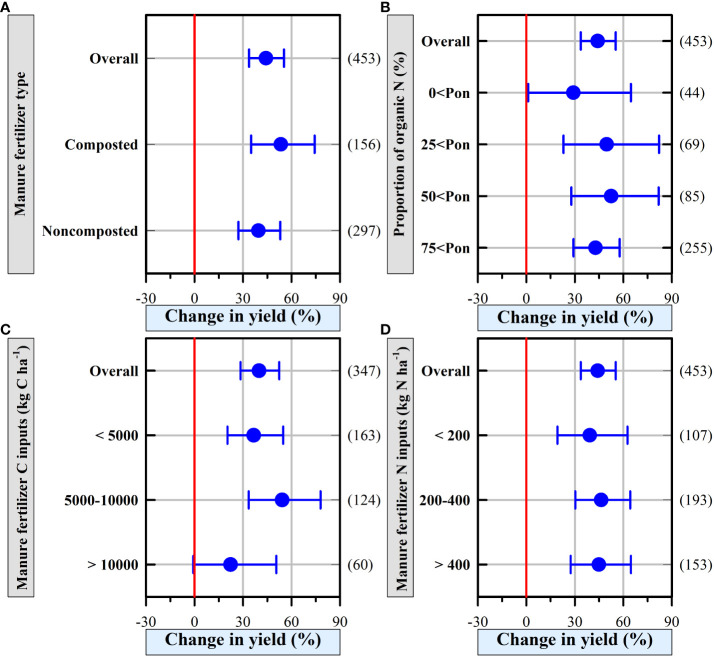
The effects of organic fertilizer properties **(A)** manure fertilizer type, **(B)** proportion of organic N, **(C)** manure fertilizer C inputs, and **(D)** manure fertilizer N inputs on greenhouse vegetable yield. The numbers of observations are shown right parentheses. The blue horizontal bars represent the 95% confidence intervals (CIs). If the CI does not overlap with 0 red line, which indicates a significant effect at *P* < 0.05.

Overall, organic fertilizer increased vegetable yield irrespective of fertilizer type (**Figure 3A**), the proportion of N (**Figure 3B**), organic fertilizer C input (**Figure 3C**), and organic fertilizer N input (**Figure 3D**), except for when the organic fertilizer C input > 10,000 kg C ha^-1^ yr^-1^ (**Figure 3C**; *p* > 0.05). In addition, a low proportion of N or organic fertilizer N input decreased the percentage change of vegetable yield and increased variances as indicated by the large CI (**Figures 3B, D**).

### Effect of organic fertilizer on vegetable yield depended on the soil properties

**Figure 4:**
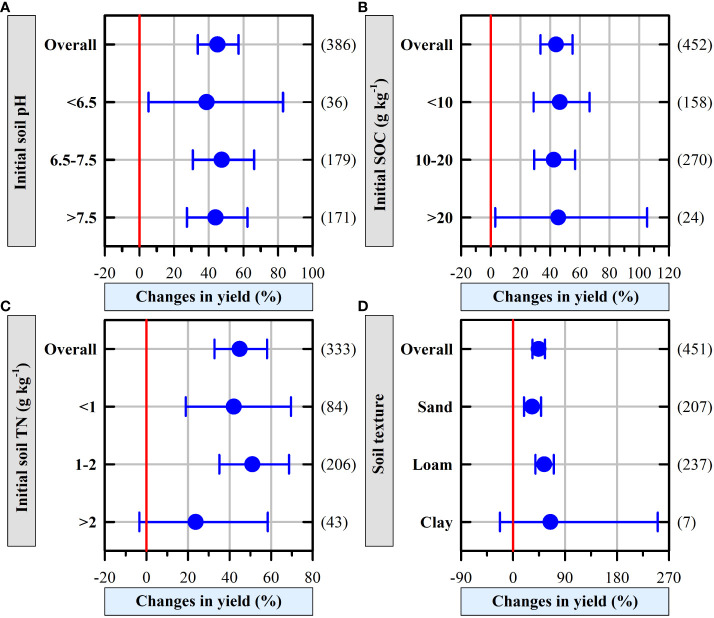
The effects of soil properties **(A)** initial soil pH, **(B)** initial SOC, **(C)** initial soil TN, and **(D)** soil texture on greenhouse vegetable yield. The numbers of observations are shown right parentheses. The blue horizontal bars represent the 95% confidence intervals (CIs). If the CI does not overlap with 0 red line, which indicates a significant effect at *P* < 0.05.

Organic fertilizer increased vegetable yield regardless of initial soil pH, while low pH decreased the percentage change of vegetable yield and increased the variances as indicated by the large CI (**Figure 4A**). Organic fertilizer increased the yields of vegetables regardless of the initial SOC content, and high initial SOC content increased the variances (CI: 3.04% to 105.34%, **Figure 4B**). Similarly, organic fertilizer increased the vegetable yield irrespective of the initial TN content except for high initial TN content, which decreased the percentage change of vegetable yield and increased variances as indicated by the large CI in **Figure 4C**. Organic fertilizer increased vegetable yield irrespective of soil texture except for clay soils, which increased the percentage change of vegetable yield and variances (CI: -22.56% to 250.68%; **Figure 4D**).

### Impact factors for the response of vegetable yields to organic fertilizer application

**Figure 5:**
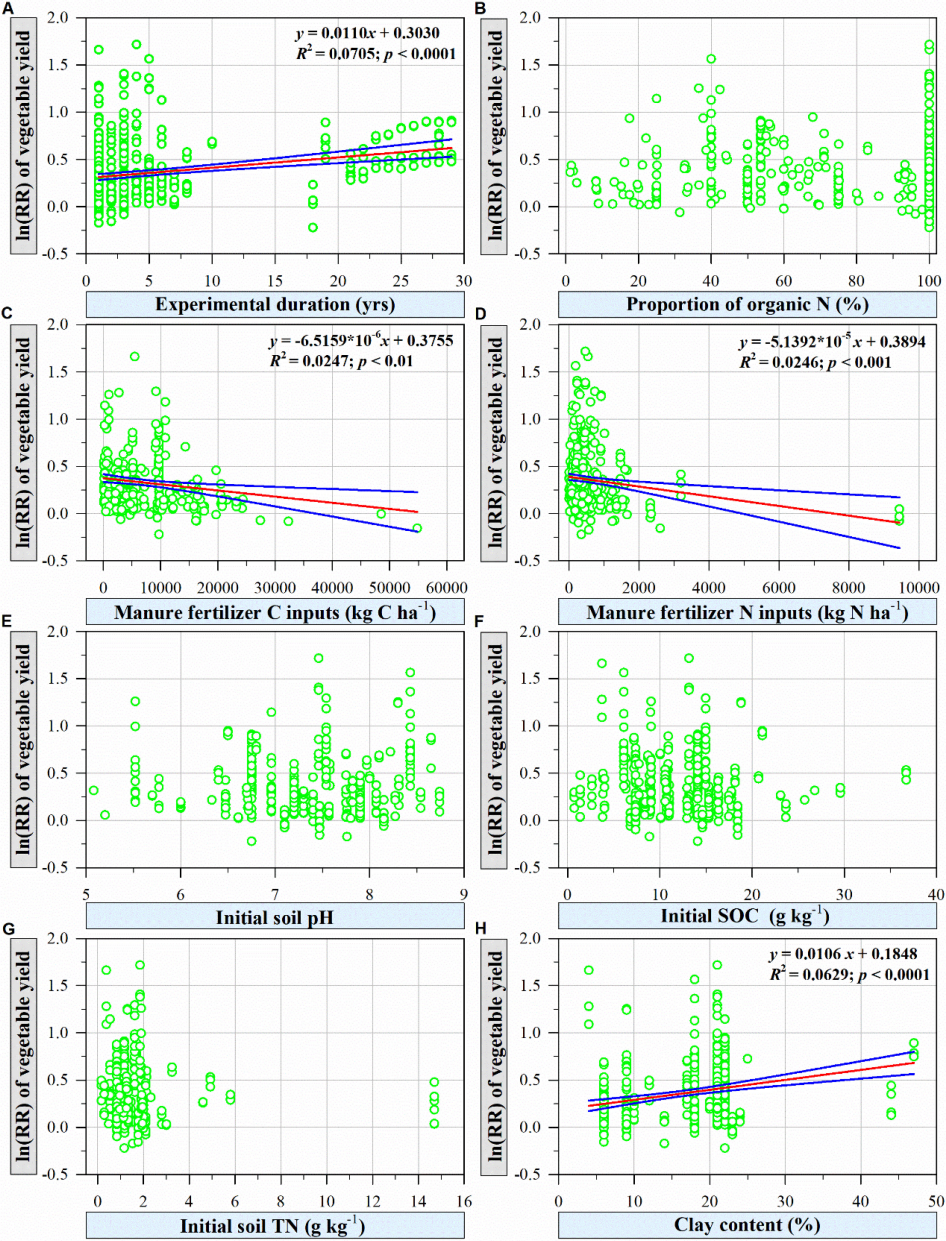
Relationships between the ln(RR) of vegetable yield under organic fertilization compared to that under no fertilization with different experimental conditions [**(A)**, experimental duration; **(B)**, proportion of organic N], organic fertilizer properties **(C)**, manure fertilizer C inputs; **(D)**, manure fertilizer N inputs) and soil properties [**(E)**, initial soil pH; **(F)**, initial SOC; **(G)**, initial soil TN, and **(H)**, soil clay content]. The red fitted lines show the OLS regression, and the areas between the two blue lines represent the 95% confidence intervals of the fitted regression model.

The response ratio of vegetable yield was significantly and positively correlated with the experimental duration (**Figure 5A**; *p*<0.0001) and soil clay content (**Figure 5H**; *p*<0.0001). Additionally, organic fertilizer C inputs (**Figure 5C**; *p*<0.01) and organic fertilizer N inputs (**Figure 5D**; *p*<0.001) were significantly negatively correlated with the response ratio of vegetable yield. In contrast, no statistically significant relationships between the response ratio of vegetable yield and the Pon (**Figure 5B**), initial soil pH (**Figure 5E**), initial SOC (**Figure 5F**), or initial soil TN (**Figure 5G**) were identified.

**Figure 6:**
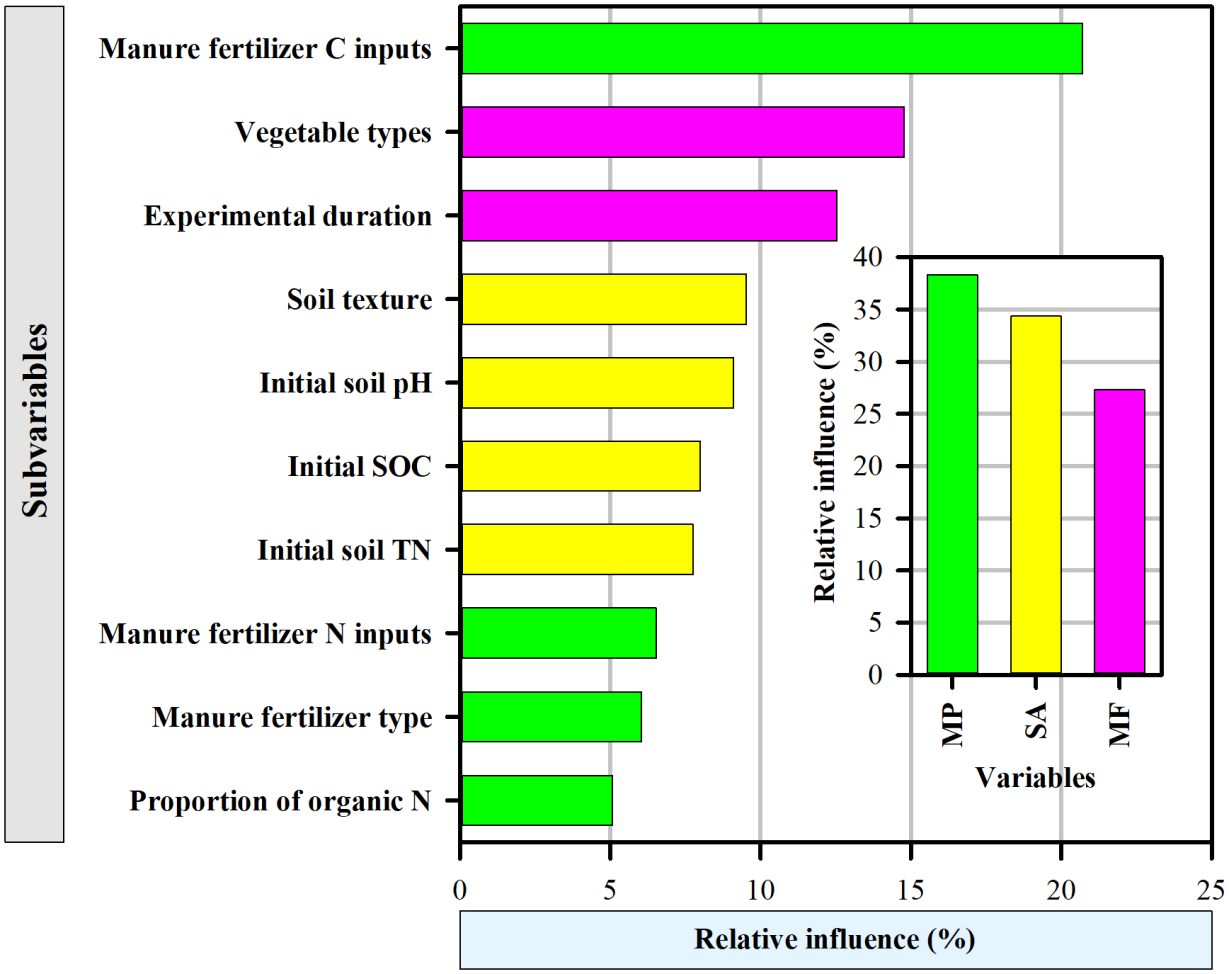
The relative influence (%) of organic fertilizer properties (*i.e.*, organic fertilizer C inputs, proportion of organic N, organic fertilizer type, organic fertilizer N inputs), soil properties (*i.e.*, initial soil total nitrogen, initial soil organic carbon, initial soil pH, soil texture) and managerial factors (*i.e.*, experimental duration, vegetable types) on vegetable yield based on aggregated boosted tree (ABT) model analysis. Figure abbreviations denote manure properties (MP), soil properties (SA), managerial factors (MF).

ABT analysis was applied to compare the relative importance of experimental conditions, organic fertilizer properties and edaphic factors on the response ratio of vegetable yield (**Figure 6**). In total, 82.39% of the variance in vegetable yield could be explained by the first seven factors. Organic fertilizer C inputs, vegetable type and experimental duration were particularly important in explaining the variation in vegetable yield and accounted for approximately 48.02% of the total variation. Moreover, the organic fertilizer C inputs were the most influential variable on the vegetable yield (>20%) among the 10 chosen variables. In total, the organic fertilizer properties (38.32%, including organic fertilizer C inputs, organic fertilizer N inputs, organic fertilizer type, and proportion of organic N), contributed the most to the variance in the response of vegetable yield to organic fertilizer, followed by edaphic factors (34.37%, including soil texture, initial soil pH, initial SOC, and initial soil TN) and experimental conditions (27.31%, including experimental duration, and vegetable type).

## Discussion

Our meta-analysis of 68 studies revealed that organic fertilizer application significantly increased the vegetable yield in greenhouse vegetable systems by 44.11% in China, compared with no fertilizer applied. Although the magnitude of the vegetable yield response to organic fertilizer application varied with the vegetable type, experimental duration, organic fertilizer type and initial soil properties across the nation, our results indicated that the application of farmyard manure would be a promising strategy for increasing vegetable yield in greenhouse vegetable systems in China.

### Vegetable type responses to organic fertilizer

Overall, the current meta-analysis demonstrated that organic fertilizer application consistently increased vegetable yield in greenhouse vegetable systems in China. Organic fertilizer application enhanced more leafy and fruit vegetable yields than stem vegetables by 76.44% and 41.75%, respectively (**Figure 2A**). The reason is likely that vegetables have shallow root systems and low root density, and thus, N input can facilitate their growth (Xu et al., 2020), particularly leafy and fruit vegetables instead of stem vegetables (Yahia et al., 2019; Huang et al., 2020). While, stem vegetables, e.g., celery, also need phosphorus to relieve nutrient limitation that causes lower root and aboveground biomass (Matysek et al., 2019). For instance, a field trial in Australia reported that chicken manure alone significantly lowered celery yield (Suter et al., 2021); which is likely because the N from manure is slowly mineralized, and the N supply does not match the N demand for celery. Thus, the application of organic fertilizer should be widely encouraged in producing leafy vegetables and fruit vegetables, and further study is needed to figure out optimal fertilizer management in increasing stem vegetable yield in China.

Vegetable yield in greenhouse vegetable systems increased with the extension of experimental duration (**Figure 5A**); a similar result was observed by Bonanomi et al. (2014), who found that organic amendment positively affected microbiological and enzymatic parameters, and improved soil quality, thus, eventually facilitated vegetables grown under plastic tunnels in the long run.

### Effect of organic fertilizer type and application rate on vegetable yields

Although our analysis found that organic fertilizer type, whether composted or non-composted organic fertilizer, had no different effects on the yields of vegetables (**Figure 3A**), producers are encouraged to apply composted organic fertilizer because high-temperature (> 60°C) composting can effectively decrease the viability of worm eggs, pathogens and weed seeds (Ichihashi et al., 2020), and thus, protect vegetables from pests and pathogens and reduce the competition between weeds and vegetables for nutrients, moisture and light resources.

Organic fertilizer application increased the vegetable yields in greenhouse vegetable systems, irrespective of the proportion of organic N (**Figure 3B**), while vegetable yields at moderate Pon levels (50%<Pon ≤ 75%) were slightly higher than those at lower (0%<Pon ≤ 25%) and higher Pon levels (75%<Pon ≤ 100%). These results are in line with the study of Zhou et al. (2019a), who found that organic fertilizer treatments improved vegetable yields, yet vegetable yields at rates of organic N of 33%≤Pon ≤ 66% were significantly higher than those of other treatments, and this was largely affected by nonlinear changes in N use efficiency. In addition, the highest vegetable yields were found following the use of organic fertilizer and synthetic N fertilizer together for more than 10 consecutive years, and these high yields were principally owing to the interactions between the organic fertilizer and the synthetic N fertilizers (Chivenge et al., 2011; Cai et al., 2019). This may be because most of the N in organic fertilizers was relatively less available for vegetable uptake than that in synthetic fertilizers (Sileshi et al., 2019).

Excessive C input application rate (>10000 kg C ha^-1^) resulting from the organic fertilizer addition did not increase the vegetable yields (**Figure 3C**). This is probably because high C input rates from organic fertilizer increase the soil C:N ratio, which leads to a decrease in N mineralization (Probert et al., 2005). Consequently, soils with higher C:N ratios cannot provide sufficient available N to satisfy the needs of vegetable growth during the vegetable growing season (Xia et al., 2017; Zhou et al., 2019a). In contrast, organic fertilizer application increased vegetable yield in greenhouse vegetable systems regardless of the N input application rate (**Figure 3D**), whereas excessive organic fertilizer N inputs did not result in a further increase in vegetable yield. This is likely that N use efficiency decreases with the increasing amount of N supplied *via* organic fertilizer (Zhou et al., 2019a), and vegetable yield is positively related to N use efficiency rather than the amount of N input (Zhu et al., 2005). With excessive N inputs from manure and mineral fertilizers, the production of protons through the nitrification process induced soil acidification in greenhouse vegetable systems (Bai et al., 2020), and soil acidification dramatically affects crop yield (Li et al., 2019b), since soil acidification reduces the availability of several vital nutrient elements (e.g., phosphorous), while exacerbating the toxicity level of others (e.g., aluminium) by altering numerous chemical and biological reactions in the soil (Yang et al., 2018).

### Effect of soil factors on vegetable yields

Organic fertilizer application increased vegetable yield in greenhouse vegetable systems regardless of the initial soil pH (**Figure 4A**). While the potential mechanism of increasing vegetable yield with organic fertilizer might vary with the initial soil pH. Organic fertilizer increased vegetable yield in greenhouse vegetable systems mainly by increasing SOC (Wang et al., 2019a), soil pH (Du et al., 2020), and soil available nutrients (e.g., available N, phosphorus, or potassium) (Qaswar et al., 2020) in acidic soils. In alkaline soils, organic fertilizer did not significantly affect the soil pH (Herencia et al., 2007); while it greatly increased phosphorus transformation (Zhao et al., 2019), potassium availability (Chen et al., 2020), the relative abundance of beneficial bacteria (Zhao et al., 2019), and soil C-cycling enzyme activity (Luan et al., 2019; Miao et al., 2019).

SOC generally plays an important role in maintaining soil fertility and vegetable yields (Wang et al., 2019a). A previous study showed that a suitable initial SOC in greenhouse vegetable systems contributes to high vegetable yield (Morra et al., 2010) because SOC can contribute to enhancing soil porosity and water-holding capacity (Li et al., 2019c); these effects result in greater soil N mineralization, microbial activity, and microbial biomass (Li et al., 2018), which effectively promote vegetable growth (Chen et al., 2012; Zhen et al., 2020). However, we did not find significant differences in yield among the three levels of initial SOC concentrations (**Figure 4B**). While Du et al. (2020) found that the positive responses of crop yields to manure application significantly with decreasing initial SOC concentrations. The reason for this discrepancy is likely that compared with open fields, in greenhouse vegetable systems, the application of large amounts of exogenous C *via* organic fertilizer affects vegetable growth more than that of the initial SOC (**Figure 6**) (Chen et al., 2012; Du et al., 2020).

Organic fertilizer addition did not increase the vegetable yield in greenhouse vegetable systems when the initial soil total N > 2 g kg^-1^ (**Figure 4C**). This can be potentially explained that vegetables have shallow root systems and low root densities, which makes them sensitive to N availability (Xu et al., 2020). Therefore, given the high initial soil total N concentration, additional N input from organic fertilizer would not further increase vegetable yield. This result also suggests that soil testing and formula fertilization should be determined for greenhouse vegetable production in China; this has promising potential to reduce excessive use of N fertilizer and subsequent pollution in soil and water.

Soil texture is an important factor regulating soil productivity (Ziadi et al., 2013). Thus, it is necessary to consider soil texture when applying fertilizer for agricultural production (Cambouris et al., 2016). Applying organic fertilizer in greenhouse vegetable systems promoted an increase in vegetable yield except for clayey soils (**Figure 4D**). A previous study has found that applying organic fertilizer to clayey soils can result in the accumulation of organic contaminants (Song et al., 2018) or heavy metals (Zhen et al., 2020), which impairs vegetable growth. Yazdanpanah et al. (2016) reported that organic fertilizer application increased the amount of soil stable aggregates and SOC in sandy soils and could subsequently provide a good environment for vegetable growth. Significantly higher vegetable yields were achieved when organic fertilizer was applied to loamy soils because vegetables grown in loamy soils took up more N than those grown in other types of soils (Ahmadi et al., 2011). Therefore, producers need to cultivate vegetables in consideration of soil texture in greenhouse vegetable systems.

Although this meta-analysis extracted data from studies exclusively located in China, which has the largest vegetable cultivation area in the world (Wang et al., 2019b) and land degradation issues because of inappropriate fertilizer management; based on the information on manure properties, soil conditions, and managerial factors of 453 paired observations from 68 published studies in China, the findings of this meta-analysis could provide a wider interpretation and apply those results broadly. Additionally, the small sampling size increased the variances of associated results, as indicated by the large CIs (e.g., **Figures 4C, D**), which weakens the power of this meta-analysis, and relevant interpretations should be made with caution. The lack of economic and environmental data dampens our understanding of the response of vegetable production to organic fertilizer application, compared to the control or even synthetic fertilizer, and further study is warranted.

## Conclusions

Based on the meta-analysis of 68 published peer-reviewed papers, we demonstrated that organic fertilizer application overall significantly enhanced vegetable yields by 44.11% in China, compared with no fertilizer application. This indicated that rational farmyard manure application would be a promising strategy for increasing vegetable yield in greenhouse vegetable systems in China. Furthermore, the long-term organic fertilizer application would benefit vegetable yields more. Whereas additional practices remain needed to increase vegetable yields when the vegetable is stem vegetable, the organic fertilizer C input is > 10,000 kg C ha^-1^ yr^-1^, the initial soil N concentration is > 2 g kg^-1^, or in the clayey soils in greenhouse vegetable systems in China.

## Author contributions

YX: Designed research, Collected data, Performed meta-analysis, Visualization, Writing – original draft, Funding acquisition. YLi: Designed research, Performed meta-analysis, Visualization, Writing – review & editing, Funding acquisition. XL: Writing – review. YLiu: Collected data. XX: Data curation. BY: Designed research, Supervision, Project administration, Funding acquisition. JX: Writing – review. LZ: Collected data. JF: Writing – review. XXu: Visualization. YLi: Data curation. All authors contributed to the article and approved the submitted version.

## Acknowledgments

This work was supported by grants from the Central Non-profit Research Institution of Chinese Academy of Forestry (CAFYBB2020ZE005, CAFYBB2020GD001), Guizhou Provincial Science and Technology Projects (QKHJC-ZK [2021] YB335), Starting Research Fund of Team Construction of Lanzhou University Double First-Rate project (561120207), National Key Research and Development Program of China (2020YFF0305900), Natural Science Foundation of China (32101431; 32260725). We are grateful to all reviewers who provided thoughtful comments to improve the manuscript.

## Conflict of interest

Author JX is employed by New Zealand Forest Research Institute (Scion), Christchurch, New Zealand.

The remaining authors declare that the research was conducted in the absence of any commercial or financial relationships that could be construed as a potential conflict of interest.

## Publisher’s note

All claims expressed in this article are solely those of the authors and do not necessarily represent those of their affiliated organizations, or those of the publisher, the editors and the reviewers. Any product that may be evaluated in this article, or claim that may be made by its manufacturer, is not guaranteed or endorsed by the publisher.
