# Supplementary material for: Manure properties, soil conditions and managerial factors regulate greenhouse vegetable yield with organic fertilizer application across China
Source: Front Plant Sci. 2022 Oct 21;13:1009631. doi: 10.3389/fpls.2022.1009631 (PMC9635265; doi:10.3389/fpls.2022.1009631)
Supplement: Supplementary file 1 [file Table_1.docx]

**Supplementary material**

**Manure properties, soil conditions and managerial factors regulate greenhouse vegetable yield with organic fertilizer application across China**

Yangzhou Xiang ^a, 1^, Yuan Li ^b, 1^, Xuqiang Luo ^a, 1^, Ying Liu ^c^, Xuejiao Yue ^d,e^, Bin Yao ^d,e,^^[[1]](#footnote-1)^*

, Jianming Xue ^f,g^, Leiyi Zhang ^h^, Jing Fan ^a^, Xiuyue Xu ^a^, Yonghua Li ^d,e^

*^a^* *Guizhou Provincial Key Laboratory of Geographic State Monitoring of Watershed, School of Geography and Resources, Guizhou Education University, Guiyang 550018, China*

*^b^* *State Key Laboratory of Grassland Agro-ecosystems, College of Pastoral Agriculture Science and Technology, Lanzhou University, Lanzhou 730020, China*

*^c^* *School of Biological Sciences, Guizhou Education University, Guiyang 550018, China*

*^d^* *Institute of Desertification Studies, Chinese Academy of Forestry, Beijing 100091, China*

*^e^* *Institute of Desertification Studies, Chinese Academy of Forestry, Beijing 100091, China*

*^f^ College of Biology and the Environment, Nanjing Forestry University, Nanjing, 210037, China*

*^g^ New Zealand Forest Research Institute Ltd (Scion), Scion 8440, New Zealand*

*^h^ South China Institute of Environmental Sciences, Ministry of Ecology and Environment of PRC, Guangzhou 510655, China*

^1^ Yangzhou Xiang, Yuan Li and Xuqiang Luo contributed equally to this work.

**Table S1.** List of studies included in this meta-analysis

| **References** | **Vegetable species** | **Organic fertilizer** | **Organic fertilizer C inputs**  **(kg N ha^-1^ yr^-1^)** | **Organic fertilizer N inputs**  **(kg N ha^-1^ yr^-1^)** | **Synthetic N inputs**  **(kg N ha^-1^ yr^-1^)** | **Initial pH** | **Initial SOC** | **Initial TN** | **Soil texture** |
| --- | --- | --- | --- | --- | --- | --- | --- | --- | --- |
| Zhang and Zhou, 2012 | tomato | compost | 17654.30 | 547.95 | 0.00 | 7.30 | 17.75 | 1.94 | Sand |
| Zhang and Zhou, 2012 | tomato | compost | 17295.10 | 560.40 | 0.00 | 7.30 | 17.75 | 1.94 | Sand |
| Zhang and Zhou, 2012 | tomato | compost | 16936.12 | 572.85 | 0.00 | 7.30 | 17.75 | 1.94 | Sand |
| Zhang and Zhou, 2012 | tomato | compost | 16576.68 | 585.30 | 0.00 | 7.30 | 17.75 | 1.94 | Sand |
| Zhang and Zhou, 2012 | tomato | compost | 16217.47 | 597.90 | 0.00 | 7.30 | 17.75 | 1.94 | Sand |
| Zhang and Zhou, 2012 | tomato | compost | 15858.25 | 610.35 | 0.00 | 7.30 | 17.75 | 1.94 | Sand |
| Zhang and Zhou, 2012 | tomato | compost | 15499.05 | 622.80 | 0.00 | 7.30 | 17.75 | 1.94 | Sand |
| Gao and Ge, 2005 | tomato | farmyard manure | 9700.50 | 352.50 | 0.00 | 6.48 | 13.17 |  | Loam |
| Gao and Ge, 2005 | tomato | farmyard manure | 9700.50 | 352.50 | 304.48 | 6.48 | 13.17 |  | Loam |
| Gao and Ge, 2005 | tomato | farmyard manure | 9700.50 | 352.50 | 608.97 | 6.48 | 13.17 |  | Loam |
| Gao and Ge, 2005 | tomato | farmyard manure | 9700.50 | 352.50 | 0.00 | 6.48 | 13.17 |  | Loam |
| Gao and Ge, 2005 | tomato | farmyard manure | 9700.50 | 352.50 | 0.00 | 6.48 | 13.17 |  | Loam |
| Gao and Ge, 2005 | tomato | farmyard manure | 9700.50 | 352.50 | 304.48 | 6.48 | 13.17 |  | Loam |
| Gao and Ge, 2005 | tomato | farmyard manure | 9700.50 | 352.50 | 304.48 | 6.48 | 13.17 |  | Loam |
| Gao and Ge, 2005 | tomato | farmyard manure | 9700.50 | 352.50 | 0.00 | 6.48 | 13.17 |  | Loam |
| Gao and Ge, 2005 | tomato | farmyard manure | 9700.50 | 352.50 | 304.48 | 6.48 | 13.17 |  | Loam |
| Zhou and Yang, 2013 | tomato | farmyard manure | 4544.88 | 666.00 | 59.80 | 6.96 | 8.97 | 0.54 | Loam |
| Zhou and Yang, 2013 | tomato | farmyard manure |  | 777.07 | 59.80 | 6.96 | 8.97 | 0.54 | Loam |
| Li, 2004 | cucumber | farmyard manure |  | 1470.00 | 600.00 | 7.97 | 7.31 | 3.25 | Loam |
| Li, 2004 | cucumber | farmyard manure |  | 1470.00 | 300.00 | 7.97 | 7.31 | 3.25 | Loam |
| Li, 2004 | tomato | farmyard manure |  | 1470.00 | 600.00 | 7.58 | 10.89 | 2.14 | Loam |
| Li, 2004 | tomato | farmyard manure |  | 1470.00 | 300.00 | 7.58 | 10.89 | 2.14 | Loam |
| Li, 2004 | tomato | farmyard manure |  | 1470.00 | 0.00 | 7.58 | 10.89 | 2.14 | Loam |
| Ren, 2018 | tomato | farmyard manure | 9700.50 | 352.50 | 0.00 | 6.75 | 14.09 | 1.164 | Loam |
| Ren, 2018 | tomato | farmyard manure | 9700.50 | 352.50 | 0.00 | 6.75 | 14.09 | 1.164 | Loam |
| Ren, 2018 | tomato | farmyard manure | 9700.50 | 352.50 | 304.48 | 6.75 | 14.09 | 1.164 | Loam |
| Gao, 2019 | tomato | farmyard manure |  | 565.00 | 0.00 | 7.76 | 8.87 | 1.53 | Sand |
| Gao, 2019 | tomato | farmyard manure |  | 302.00 | 0.00 | 7.76 | 8.87 | 1.53 | Sand |
| Gao, 2019 | tomato | farmyard manure |  | 565.00 | 0.00 | 7.76 | 8.87 | 1.53 | Sand |
| Gao, 2019 | tomato | farmyard manure |  | 272.00 | 0.00 | 7.76 | 8.87 | 1.53 | Sand |
| Zhang et al., 2017 | tomato | compost | 16421.70 | 960.00 | 60.67 | 6.96 | 8.97 | 0.54 | Loam |
| Zhang et al., 2017 | tomato | compost | 4545.30 | 666.00 | 60.67 | 6.96 | 8.97 | 0.54 | Loam |
| Zhang et al., 2017 | tomato | compost | 16421.70 | 960.00 | 60.67 | 6.96 | 8.97 | 0.54 | Loam |
| Zhang et al., 2017 | tomato | compost | 4545.30 | 666.00 | 60.67 | 6.96 | 8.97 | 0.54 | Loam |
| Han, 2016 | tomato | farmyard manure | 9700.50 | 352.50 | 0.00 | 6.75 | 14.09 | 1.164 | Loam |
| Han, 2016 | tomato | farmyard manure | 9700.50 | 352.50 | 0.00 | 6.75 | 14.09 | 1.164 | Loam |
| Han, 2016 | tomato | farmyard manure | 9700.50 | 352.50 | 304.48 | 6.75 | 14.09 | 1.164 | Loam |
| Han, 2016 | tomato | farmyard manure | 9700.50 | 352.50 | 304.48 | 6.75 | 14.09 | 1.164 | Loam |
| Li, 2012 | purple cabbage | farmyard manure | 5237.40 | 531.00 | 0.00 | 8.74 | 7.57 | 0.98 | Loam |
| Li, 2012 | purple cabbage | farmyard manure | 12921.24 | 651.00 | 0.00 | 8.74 | 7.57 | 0.98 | Loam |
| Li, 2012 | purple cabbage | farmyard manure | 13052.90 | 931.40 | 0.00 | 8.74 | 7.57 | 0.98 | Loam |
| Li, 2012 | purple cabbage | farmyard manure | 12882.55 | 705.80 | 0.00 | 8.74 | 7.57 | 0.98 | Loam |
| Li, 2012 | purple cabbage | farmyard manure | 13093.50 | 1327.50 | 0.00 | 8.74 | 7.57 | 0.98 | Loam |
| Zhang, 2004 | tomato | farmyard manure | 3006.59 | 224.99 | 0.00 |  | 23.66 | 2.8 | Sand |
| Zhang, 2004 | tomato | farmyard manure | 3006.59 | 224.99 | 300.00 |  | 23.66 | 2.8 | Sand |
| Zhang, 2004 | tomato | farmyard manure | 3006.59 | 224.99 | 1200.00 |  | 23.66 | 2.8 | Sand |
| Zhang, 2004 | tomato | farmyard manure | 3006.59 | 224.99 | 2400.00 |  | 23.66 | 2.8 | Sand |
| Zhang, 2004 | tomato | farmyard manure | 3006.59 | 224.99 | 0.00 |  | 23.66 | 2.8 | Sand |
| Zhang, 2004 | tomato | farmyard manure | 3006.59 | 224.99 | 300.00 |  | 23.66 | 2.8 | Sand |
| Zhang, 2004 | tomato | farmyard manure | 3006.59 | 224.99 | 1200.00 |  | 23.66 | 2.8 | Sand |
| Zhang, 2004 | tomato | farmyard manure | 3006.59 | 224.99 | 2400.00 |  | 23.66 | 2.8 | Sand |
| Tian, 2018 | tomato | farmyard manure | 9700.50 | 352.50 | 304.48 | 6.75 | 14.09 | 1.164 | Loam |
| Tian, 2018 | tomato | farmyard manure | 9700.50 | 352.50 | 0.00 | 6.75 | 14.09 | 1.164 | Loam |
| Tian, 2018 | tomato | farmyard manure | 9700.50 | 352.50 | 0.00 | 6.75 | 14.09 | 1.164 | Loam |
| Tian, 2018 | tomato | farmyard manure | 9700.50 | 352.50 | 304.48 | 6.75 | 14.09 | 1.164 | Loam |
| Tian, 2018 | tomato | farmyard manure | 9700.50 | 352.50 | 304.48 | 6.75 | 14.09 | 1.164 | Loam |
| Yao, 2012 | pepper | farmyard manure | 2083.41 | 120.00 | 120.00 |  | 6.29 | 0.83 | Loam |
| Yao, 2012 | pepper | farmyard manure | 1370.11 | 120.00 | 120.00 |  | 6.29 | 0.83 | Loam |
| Yao, 2012 | pepper | farmyard manure | 2300.82 | 120.00 | 120.00 |  | 6.29 | 0.83 | Loam |
| Yao, 2012 | pepper | farmyard manure | 1865.16 | 120.00 | 120.00 |  | 6.29 | 0.83 | Loam |
| Yao, 2012 | pepper | farmyard manure |  | 120.00 | 120.00 |  | 6.29 | 0.83 | Loam |
| Yao, 2012 | pepper | farmyard manure |  | 120.00 | 120.00 |  | 6.29 | 0.83 | Loam |
| Li et al., 2015 | cucumber | farmyard manure | 451.01 | 252.00 | 18.00 |  | 3.83 | 0.162 | Sand |
| Li et al., 2015 | cucumber | farmyard manure | 1567.04 | 180.00 | 90.00 |  | 3.83 | 0.162 | Sand |
| Li et al., 2015 | cucumber | farmyard manure | 876.96 | 120.00 | 150.00 |  | 3.83 | 0.162 | Sand |
| Liu et al., 2014 | pakchoi | compost | 68.28 | 37.50 | 112.50 |  | 18.15 | 1.03 | Sand |
| Liu et al., 2014 | pakchoi | compost | 136.56 | 75.00 | 75.00 |  | 18.15 | 1.03 | Sand |
| Liu et al., 2014 | pakchoi | compost | 204.84 | 112.50 | 37.50 |  | 18.15 | 1.03 | Sand |
| Liu et al., 2014 | pakchoi | compost | 273.12 | 150.00 | 0.00 |  | 18.15 | 1.03 | Sand |
| Wan et al., 2013 | cucumber | compost | 855.33 | 130.50 | 0.00 | 7.38 | 8.35 | 1.15 | Loam |
| Wan et al., 2013 | cucumber | compost | 982.96 | 149.98 | 74.98 | 7.38 | 8.35 | 1.15 | Loam |
| Wan et al., 2013 | cucumber | compost | 737.35 | 112.50 | 112.24 | 7.38 | 8.35 | 1.15 | Loam |
| Wan et al., 2013 | cucumber | compost | 491.75 | 75.03 | 149.96 | 7.38 | 8.35 | 1.15 | Loam |
| Cai et al., 2019 | tomato | farmyard manure | 71.15 | 93.67 | 0.00 | 8.54 | 0.71 |  | Sand |
| Cai et al., 2019 | tomato | farmyard manure | 87.77 | 98.33 | 0.00 | 8.54 | 0.71 |  | Sand |
| Cai et al., 2019 | tomato | farmyard manure | 94.54 | 91.33 | 0.00 | 8.54 | 0.71 |  | Sand |
| Cai et al., 2019 | tomato | farmyard manure | 119.09 | 112.33 | 0.00 | 8.54 | 0.71 |  | Sand |
| Huang, 2016 | tomato | farmyard manure | 20545.05 | 1200.00 | 60.67 | 6.96 | 8.97 | 0.54 | Loam |
| Huang, 2016 | tomato | farmyard manure | 5700.68 | 832.50 | 60.67 | 6.96 | 8.97 | 0.54 | Loam |
| Huang, 2016 | tomato | farmyard manure | 20545.05 | 1200.00 | 60.67 | 6.96 | 8.97 | 0.54 | Loam |
| Huang, 2016 | tomato | farmyard manure | 5700.68 | 832.50 | 60.67 | 6.96 | 8.97 | 0.54 | Loam |
| Huang, 2016 | tomato | farmyard manure | 5700.68 | 832.50 | 0.00 | 6.96 | 8.97 | 0.54 | Loam |
| Wang, 2017 | tomato | farmyard manure | 16275.00 | 9439.50 | 0.00 | 7.10 | 14.62 | 2.1 | Loam |
| Wang, 2017 | tomato | farmyard manure | 16275.00 | 9439.50 | 187.50 | 7.10 | 14.62 | 2.1 | Loam |
| Wang, 2017 | tomato | farmyard manure | 16275.00 | 9439.50 | 375.00 | 7.10 | 14.62 | 2.1 | Loam |
| Wang, 2017 | tomato | farmyard manure | 16275.00 | 9439.50 | 562.50 | 7.10 | 14.62 | 2.1 | Loam |
| Tan, 2016 | tomato | farmyard manure | 9700.50 | 352.50 | 304.48 | 6.75 | 14.09 | 1.164 | Loam |
| Tan, 2016 | tomato | farmyard manure | 9700.50 | 352.50 | 304.48 | 6.75 | 14.09 | 1.164 | Loam |
| Tan, 2016 | tomato | farmyard manure | 9700.50 | 352.50 | 0.00 | 6.75 | 14.09 | 1.164 | Loam |
| Tan, 2016 | tomato | farmyard manure | 9700.50 | 352.50 | 0.00 | 6.75 | 14.09 | 1.164 | Loam |
| Tan, 2016 | tomato | farmyard manure | 9700.50 | 352.50 | 0.00 | 6.75 | 14.09 | 1.164 | Loam |
| Tan, 2016 | tomato | farmyard manure | 9700.50 | 352.50 | 304.48 | 6.75 | 14.09 | 1.164 | Loam |
| Tan, 2016 | tomato | farmyard manure | 9700.50 | 352.50 | 304.48 | 6.75 | 14.09 | 1.164 | Loam |
| Tan, 2016 | tomato | farmyard manure | 9700.50 | 352.50 | 0.00 | 6.75 | 14.09 | 1.164 | Loam |
| Tan, 2016 | tomato | farmyard manure | 9700.50 | 352.50 | 0.00 | 6.75 | 14.09 | 1.164 | Loam |
| Tan, 2016 | tomato | farmyard manure | 9700.50 | 352.50 | 0.00 | 6.75 | 14.09 | 1.164 | Loam |
| Tan, 2016 | tomato | farmyard manure | 9700.50 | 352.50 | 304.48 | 6.75 | 14.09 | 1.164 | Loam |
| Tan, 2016 | tomato | farmyard manure | 9700.50 | 352.50 | 304.48 | 6.75 | 14.09 | 1.164 | Loam |
| Tan, 2016 | tomato | farmyard manure | 9700.50 | 352.50 | 0.00 | 6.75 | 14.09 | 1.164 | Loam |
| Tan, 2016 | tomato | farmyard manure | 9700.50 | 352.50 | 0.00 | 6.75 | 14.09 | 1.164 | Loam |
| Tan, 2016 | tomato | farmyard manure | 9700.50 | 352.50 | 0.00 | 6.75 | 14.09 | 1.164 | Loam |
| Tan, 2016 | tomato | farmyard manure | 9700.50 | 352.50 | 304.48 | 6.75 | 14.09 | 1.164 | Loam |
| Tan, 2016 | tomato | farmyard manure | 9700.50 | 352.50 | 304.48 | 6.75 | 14.09 | 1.164 | Loam |
| Tan, 2016 | tomato | farmyard manure | 9700.50 | 352.50 | 0.00 | 6.75 | 14.09 | 1.164 | Loam |
| Tan, 2016 | tomato | farmyard manure | 9700.50 | 352.50 | 0.00 | 6.75 | 14.09 | 1.164 | Loam |
| Tan, 2016 | tomato | farmyard manure | 9700.50 | 352.50 | 0.00 | 6.75 | 14.09 | 1.164 | Loam |
| Tan, 2016 | tomato | farmyard manure | 9700.50 | 352.50 | 304.48 | 6.75 | 14.09 | 1.164 | Loam |
| Tan, 2016 | tomato | farmyard manure | 9700.50 | 352.50 | 304.48 | 6.75 | 14.09 | 1.164 | Loam |
| Tan, 2016 | tomato | farmyard manure | 9700.50 | 352.50 | 0.00 | 6.75 | 14.09 | 1.164 | Loam |
| Tan, 2016 | tomato | farmyard manure | 9700.50 | 352.50 | 0.00 | 6.75 | 14.09 | 1.164 | Loam |
| Tan, 2016 | tomato | farmyard manure | 9700.50 | 352.50 | 0.00 | 6.75 | 14.09 | 1.164 | Loam |
| Tan, 2016 | tomato | farmyard manure | 9700.50 | 352.50 | 304.48 | 6.75 | 14.09 | 1.164 | Loam |
| Tan, 2016 | tomato | farmyard manure | 9700.50 | 352.50 | 304.48 | 6.75 | 14.09 | 1.164 | Loam |
| Tan, 2016 | tomato | farmyard manure | 9700.50 | 352.50 | 0.00 | 6.75 | 14.09 | 1.164 | Loam |
| Tan, 2016 | tomato | farmyard manure | 9700.50 | 352.50 | 0.00 | 6.75 | 14.09 | 1.164 | Loam |
| Tan, 2016 | tomato | farmyard manure | 9700.50 | 352.50 | 0.00 | 6.75 | 14.09 | 1.164 | Loam |
| Tan, 2016 | tomato | farmyard manure | 9700.50 | 352.50 | 304.48 | 6.75 | 14.09 | 1.164 | Loam |
| Tan, 2016 | tomato | farmyard manure | 9700.50 | 352.50 | 304.48 | 6.75 | 14.09 | 1.164 | Loam |
| Tan, 2016 | tomato | farmyard manure | 9700.50 | 352.50 | 0.00 | 6.75 | 14.09 | 1.164 | Loam |
| Tan, 2016 | tomato | farmyard manure | 9700.50 | 352.50 | 0.00 | 6.75 | 14.09 | 1.164 | Loam |
| Tan, 2016 | tomato | farmyard manure | 9700.50 | 352.50 | 0.00 | 6.75 | 14.09 | 1.164 | Loam |
| Tan, 2016 | tomato | farmyard manure | 9700.50 | 352.50 | 304.48 | 6.75 | 14.09 | 1.164 | Loam |
| Tan, 2016 | tomato | farmyard manure | 9700.50 | 352.50 | 304.48 | 6.75 | 14.09 | 1.164 | Loam |
| Tan, 2016 | tomato | farmyard manure | 9700.50 | 352.50 | 0.00 | 6.75 | 14.09 | 1.164 | Loam |
| Tan, 2016 | tomato | farmyard manure | 9700.50 | 352.50 | 0.00 | 6.75 | 14.09 | 1.164 | Loam |
| Tan, 2016 | tomato | farmyard manure | 9700.50 | 352.50 | 0.00 | 6.75 | 14.09 | 1.164 | Loam |
| Tan, 2016 | tomato | farmyard manure | 9700.50 | 352.50 | 304.48 | 6.75 | 14.09 | 1.164 | Loam |
| Tan, 2016 | tomato | farmyard manure | 9700.50 | 352.50 | 304.48 | 6.75 | 14.09 | 1.164 | Loam |
| Tan, 2016 | tomato | farmyard manure | 9700.50 | 352.50 | 0.00 | 6.75 | 14.09 | 1.164 | Loam |
| Tan, 2016 | tomato | farmyard manure | 9700.50 | 352.50 | 0.00 | 6.75 | 14.09 | 1.164 | Loam |
| Tan, 2016 | tomato | farmyard manure | 9700.50 | 352.50 | 0.00 | 6.75 | 14.09 | 1.164 | Loam |
| Ge et al., 2004 | tomato | farmyard manure | 9700.50 | 352.50 | 0.00 | 6.75 | 14.09 | 1.164 | Loam |
| Ge et al., 2004 | tomato | farmyard manure | 9700.50 | 352.50 | 304.48 | 6.75 | 14.09 | 1.164 | Loam |
| Ge et al., 2004 | tomato | farmyard manure | 9700.50 | 352.50 | 608.97 | 6.75 | 14.09 | 1.164 | Loam |
| Ge et al., 2004 | tomato | farmyard manure | 9700.50 | 352.50 | 304.48 | 6.75 | 14.09 | 1.164 | Loam |
| Ge et al., 2004 | tomato | farmyard manure | 9700.50 | 352.50 | 304.48 | 6.75 | 14.09 | 1.164 | Loam |
| Zhao et al., 2010 | pakchoi | compost | 913.50 | 90.00 | 0.00 |  | 3.71 | 0.38 | Sand |
| Zhao et al., 2010 | pakchoi | compost | 2740.50 | 270.00 | 0.00 |  | 3.71 | 0.38 | Sand |
| Zhao et al., 2010 | pakchoi | compost | 5481.00 | 540.00 | 0.00 |  | 3.71 | 0.38 | Sand |
| Shen et al., 2012 | tomato | farmyard manure |  | 1014.27 | 785.73 | 8.65 | 7.19 | 0.83 | Loam |
| Shen et al., 2012 | tomato | farmyard manure |  | 503.90 | 396.10 | 8.65 | 7.19 | 0.83 | Loam |
| Shen et al., 2012 | tomato | farmyard manure |  | 900.00 | 0.00 | 8.65 | 7.19 | 0.83 | Loam |
| Zhang et al., 2019a | tomato | farmyard manure | 250.02 | 112.50 | 337.50 | 6.96 | 8.97 | 0.54 | Loam |
| Zhang et al., 2019a | tomato | farmyard manure | 500.04 | 225.00 | 225.00 | 6.96 | 8.97 | 0.54 | Loam |
| Zhang et al., 2019a | tomato | farmyard manure | 1002.24 | 450.00 | 0.00 | 6.96 | 8.97 | 0.54 | Loam |
| Luan et al., 2020 | celery | farmyard manure | 1130.00 | 112.50 | 337.50 | 7.90 | 15.30 |  | Sand |
| Luan et al., 2020 | celery | farmyard manure | 2260.00 | 225.00 | 225.00 | 7.90 | 15.30 |  | Sand |
| Luan et al., 2020 | celery | farmyard manure | 3390.00 | 337.50 | 112.50 | 7.90 | 15.30 |  | Sand |
| Luan et al., 2020 | tomato | farmyard manure | 1130.00 | 112.50 | 337.50 | 7.90 | 15.30 |  | Sand |
| Luan et al., 2020 | tomato | farmyard manure | 2260.00 | 225.00 | 225.00 | 7.90 | 15.30 |  | Sand |
| Luan et al., 2020 | tomato | farmyard manure | 3390.00 | 337.50 | 112.50 | 7.90 | 15.30 |  | Sand |
| Luan et al., 2020 | celery | farmyard manure | 1130.00 | 112.50 | 337.50 | 7.90 | 15.30 |  | Sand |
| Luan et al., 2020 | celery | farmyard manure | 2260.00 | 225.00 | 225.00 | 7.90 | 15.30 |  | Sand |
| Luan et al., 2020 | celery | farmyard manure | 3390.00 | 337.50 | 112.50 | 7.90 | 15.30 |  | Sand |
| Luan et al., 2020 | tomato | farmyard manure | 1130.00 | 112.50 | 337.50 | 7.90 | 15.30 |  | Sand |
| Luan et al., 2020 | tomato | farmyard manure | 2260.00 | 225.00 | 225.00 | 7.90 | 15.30 |  | Sand |
| Luan et al., 2020 | tomato | farmyard manure | 3390.00 | 337.50 | 112.50 | 7.90 | 15.30 |  | Sand |
| Luan et al., 2020 | celery | farmyard manure | 1130.00 | 112.50 | 337.50 | 7.90 | 15.30 |  | Sand |
| Luan et al., 2020 | celery | farmyard manure | 2260.00 | 225.00 | 225.00 | 7.90 | 15.30 |  | Sand |
| Luan et al., 2020 | celery | farmyard manure | 3390.00 | 337.50 | 112.50 | 7.90 | 15.30 |  | Sand |
| Luan et al., 2020 | tomato | farmyard manure | 1130.00 | 112.50 | 337.50 | 7.90 | 15.30 |  | Sand |
| Luan et al., 2020 | tomato | farmyard manure | 2260.00 | 225.00 | 225.00 | 7.90 | 15.30 |  | Sand |
| Luan et al., 2020 | tomato | farmyard manure | 3390.00 | 337.50 | 112.50 | 7.90 | 15.30 |  | Sand |
| Luan et al., 2020 | celery | farmyard manure | 1130.00 | 112.50 | 337.50 | 7.90 | 15.30 |  | Sand |
| Luan et al., 2020 | celery | farmyard manure | 2260.00 | 225.00 | 225.00 | 7.90 | 15.30 |  | Sand |
| Luan et al., 2020 | celery | farmyard manure | 3390.00 | 337.50 | 112.50 | 7.90 | 15.30 |  | Sand |
| Luan et al., 2020 | tomato | farmyard manure | 1130.00 | 112.50 | 337.50 | 7.90 | 15.30 |  | Sand |
| Luan et al., 2020 | tomato | farmyard manure | 2260.00 | 225.00 | 225.00 | 7.90 | 15.30 |  | Sand |
| Luan et al., 2020 | tomato | farmyard manure | 3390.00 | 337.50 | 112.50 | 7.90 | 15.30 |  | Sand |
| Luan et al., 2020 | celery | farmyard manure | 1130.00 | 112.50 | 337.50 | 7.90 | 15.30 |  | Sand |
| Luan et al., 2020 | celery | farmyard manure | 2260.00 | 225.00 | 225.00 | 7.90 | 15.30 |  | Sand |
| Luan et al., 2020 | celery | farmyard manure | 3390.00 | 337.50 | 112.50 | 7.90 | 15.30 |  | Sand |
| Luan et al., 2020 | tomato | farmyard manure | 1130.00 | 112.50 | 337.50 | 7.90 | 15.30 |  | Sand |
| Luan et al., 2020 | tomato | farmyard manure | 2260.00 | 225.00 | 225.00 | 7.90 | 15.30 |  | Sand |
| Luan et al., 2020 | tomato | farmyard manure | 3390.00 | 337.50 | 112.50 | 7.90 | 15.30 |  | Sand |
| Luan et al., 2020 | celery | farmyard manure | 1130.00 | 112.50 | 337.50 | 7.90 | 15.30 |  | Sand |
| Luan et al., 2020 | celery | farmyard manure | 2260.00 | 225.00 | 225.00 | 7.90 | 15.30 |  | Sand |
| Luan et al., 2020 | celery | farmyard manure | 3390.00 | 337.50 | 112.50 | 7.90 | 15.30 |  | Sand |
| Luan et al., 2020 | tomato | farmyard manure | 1130.00 | 112.50 | 337.50 | 7.90 | 15.30 |  | Sand |
| Luan et al., 2020 | tomato | farmyard manure | 2260.00 | 225.00 | 225.00 | 7.90 | 15.30 |  | Sand |
| Luan et al., 2020 | tomato | farmyard manure | 3390.00 | 337.50 | 112.50 | 7.90 | 15.30 |  | Sand |
| Luan et al., 2020 | celery | farmyard manure | 1130.00 | 112.50 | 337.50 | 7.90 | 15.30 |  | Sand |
| Luan et al., 2020 | celery | farmyard manure | 2260.00 | 225.00 | 225.00 | 7.90 | 15.30 |  | Sand |
| Luan et al., 2020 | celery | farmyard manure | 3390.00 | 337.50 | 112.50 | 7.90 | 15.30 |  | Sand |
| Luan et al., 2020 | tomato | farmyard manure | 1130.00 | 112.50 | 337.50 | 7.90 | 15.30 |  | Sand |
| Luan et al., 2020 | tomato | farmyard manure | 2260.00 | 225.00 | 225.00 | 7.90 | 15.30 |  | Sand |
| Luan et al., 2020 | tomato | farmyard manure | 3390.00 | 337.50 | 112.50 | 7.90 | 15.30 |  | Sand |
| Luan et al., 2020 | celery | farmyard manure | 1130.00 | 112.50 | 337.50 | 7.90 | 15.30 |  | Sand |
| Luan et al., 2020 | celery | farmyard manure | 2260.00 | 225.00 | 225.00 | 7.90 | 15.30 |  | Sand |
| Luan et al., 2020 | celery | farmyard manure | 3390.00 | 337.50 | 112.50 | 7.90 | 15.30 |  | Sand |
| Luan et al., 2020 | tomato | farmyard manure | 1130.00 | 112.50 | 337.50 | 7.90 | 15.30 |  | Sand |
| Luan et al., 2020 | tomato | farmyard manure | 2260.00 | 225.00 | 225.00 | 7.90 | 15.30 |  | Sand |
| Luan et al., 2020 | tomato | farmyard manure | 3390.00 | 337.50 | 112.50 | 7.90 | 15.30 |  | Sand |
| Zhang et al., 2018 | tomato | farmyard manure | 250.02 | 112.50 | 337.50 | 6.96 | 8.97 | 0.54 | Loam |
| Zhang et al., 2018 | tomato | farmyard manure | 1002.24 | 450.00 | 0.00 | 6.96 | 8.97 | 0.54 | Loam |
| Feng et al., 2015 | celery | farmyard manure | 186.75 | 5.63 | 0.00 | 8.31 | 2.62 |  | Loam |
| Feng et al., 2015 | celery | farmyard manure | 186.75 | 5.63 | 180.00 | 8.31 | 2.62 |  | Loam |
| Feng et al., 2015 | celery | farmyard manure | 186.75 | 5.63 | 270.00 | 8.31 | 2.62 |  | Loam |
| Feng et al., 2015 | celery | farmyard manure | 186.75 | 5.63 | 360.00 | 8.31 | 2.62 |  | Loam |
| Feng et al., 2015 | celery | farmyard manure | 186.75 | 5.63 | 450.00 | 8.31 | 2.62 |  | Loam |
| Zhang, 2018 | tomato | compost | 20475.00 | 1200.00 | 58.50 | 6.96 | 8.97 | 0.54 | Loam |
| Zhang, 2018 | tomato | farmyard manure | 5662.50 | 832.50 | 58.50 | 6.96 | 8.97 | 0.54 | Loam |
| Lv et al., 2018 | cucumber | farmyard manure |  | 21.13 | 232.00 | 7.21 | 8.24 | 1.45 | Loam |
| Lv et al., 2018 | cucumber | farmyard manure |  | 21.13 | 232.00 | 7.21 | 8.24 | 1.45 | Loam |
| Lv et al., 2018 | cucumber | farmyard manure |  | 21.13 | 232.00 | 7.21 | 8.24 | 1.45 | Loam |
| Hu, 2015 | eggplant | farmyard manure | 3023.94 | 126.37 | 276.75 | 8.15 | 6.80 | 1.18 | Sand |
| Hu, 2015 | eggplant | farmyard manure | 3407.22 | 192.79 | 276.75 | 8.15 | 6.80 | 1.18 | Sand |
| Hu, 2015 | eggplant | farmyard manure | 4637.49 | 285.24 | 276.75 | 8.15 | 6.80 | 1.18 | Sand |
| Hu, 2015 | eggplant | farmyard manure | 11529.69 | 418.97 | 276.75 | 8.15 | 6.80 | 1.18 | Sand |
| Hu, 2015 | eggplant | farmyard manure | 17597.39 | 627.66 | 276.75 | 8.15 | 6.80 | 1.18 | Sand |
| Hu, 2015 | eggplant | farmyard manure | 8527.88 | 637.31 | 276.75 | 8.15 | 6.80 | 1.18 | Sand |
| Zhao et al., 2009 | cucumber | compost |  | 300.00 | 0.00 | 6.00 | 8.41 | 1.13 | Loam |
| Zhao et al., 2009 | cucumber | compost |  | 240.00 | 60.00 | 6.00 | 8.41 | 1.13 | Loam |
| Zhao et al., 2009 | cucumber | compost |  | 180.00 | 120.00 | 6.00 | 8.41 | 1.13 | Loam |
| Zhao et al., 2009 | cucumber | compost |  | 120.00 | 180.00 | 6.00 | 8.41 | 1.13 | Loam |
| Zhao et al., 2009 | cucumber | compost |  | 60.00 | 240.00 | 6.00 | 8.41 | 1.13 | Loam |
| Xu et al., 2004 | cucumber | compost |  | 948.60 | 0.00 |  | 3.78 | 0.484 | Sand |
| Duan, 2016 | tomato | farmyard manure | 9439.50 | 2325.00 | 0.00 | 7.10 | 14.62 | 2.1 | Loam |
| Duan, 2016 | tomato | farmyard manure | 9439.50 | 2325.00 | 0.00 | 7.10 | 14.62 | 2.1 | Loam |
| Duan, 2016 | tomato | farmyard manure | 9439.50 | 2325.00 | 187.50 | 7.10 | 14.62 | 2.1 | Loam |
| Duan, 2016 | tomato | farmyard manure | 9439.50 | 2325.00 | 375.00 | 7.10 | 14.62 | 2.1 | Loam |
| Duan, 2016 | tomato | farmyard manure | 9439.50 | 2325.00 | 562.50 | 7.10 | 14.62 | 2.1 | Loam |
| Yang et al., 2013 | tomato | farmyard manure | 13793.85 | 525.00 | 0.00 | 6.71 | 12.98 | 1.01 | Loam |
| Yang et al., 2013 | tomato | compost | 14946.60 | 885.00 | 0.00 | 6.71 | 12.98 | 1.01 | Loam |
| Luo et al., 2015 | pepper | farmyard manure | 845.35 | 112.00 | 0.00 | 6.40 | 36.71 | 4.92 | Loam |
| Luo et al., 2015 | pepper | farmyard manure | 846.38 | 112.00 | 0.00 | 6.40 | 36.71 | 4.92 | Loam |
| Luo et al., 2015 | pepper | farmyard manure | 2265.00 | 112.00 | 0.00 | 6.40 | 36.71 | 4.92 | Loam |
| Liu et al., 2019 | cucumber | compost | 4346.69 | 906.00 | 0.00 | 8.10 | 8.70 |  | Sand |
| Liu et al., 2019 | cucumber | compost | 4346.69 | 906.00 | 300.00 | 8.10 | 8.70 |  | Sand |
| Liu et al., 2019 | cucumber | compost | 4346.69 | 906.00 | 675.00 | 8.10 | 8.70 |  | Sand |
| Zhao et al., 2014 | tomato | compost | 5943.42 | 268.87 | 0.00 | 5.20 | 8.35 |  | Loam |
| Wu et al., 2018 | tomato | compost | 1196.00 | 133.50 | 133.50 | 7.90 | 8.87 |  | Sand |
| Wu et al., 2018 | tomato | compost | 792.85 | 88.50 | 178.50 | 7.90 | 8.87 |  | Sand |
| Wu et al., 2018 | tomato | compost | 1599.14 | 178.50 | 88.50 | 7.90 | 8.87 |  | Sand |
| Zhou and Luo, 2004 | pakchoi | compost |  | 112.50 | 112.50 | 5.70 | 23.08 | 4.6 |  |
| Zhou and Luo, 2004 | spinach | compost |  | 112.50 | 112.50 | 5.70 | 23.08 | 4.6 |  |
| Li et al., 2016 | tomato | farmyard manure | 9700.50 | 352.50 | 0.00 | 6.75 | 14.09 | 1.164 | Loam |
| Li et al., 2016 | tomato | farmyard manure | 9700.50 | 352.50 | 143.49 | 6.75 | 14.09 | 1.164 | Loam |
| Li et al., 2016 | tomato | farmyard manure | 9700.50 | 352.50 | 286.99 | 6.75 | 14.09 | 1.164 | Loam |
| Luo et al., 2016 | cucumber | farmyard manure | 564.97 | 56.22 | 56.22 | 6.70 | 29.56 | 5.8 | Loam |
| Luo et al., 2016 | cucumber | farmyard manure | 424.33 | 56.22 | 56.22 | 6.70 | 29.56 | 5.8 | Loam |
| Luo et al., 2016 | cucumber | farmyard manure | 633.73 | 56.22 | 56.22 | 6.70 | 29.56 | 5.8 | Loam |
| Song et al., 2017b | pepper | commercial organic fertilizer | 19670.70 | 1659.00 | 0.00 | 7.40 | 16.36 | 2 | Sand |
| Song et al., 2017b | pepper | commercial organic fertilizer | 14753.03 | 1244.25 | 0.00 | 7.40 | 16.36 | 2 | Sand |
| Song et al., 2017b | pepper | commercial organic fertilizer | 9835.35 | 829.50 | 0.00 | 7.40 | 16.36 | 2 | Sand |
| Song et al., 2017b | pepper | commercial organic fertilizer | 9835.35 | 829.50 | 273.33 | 7.40 | 16.36 | 2 | Sand |
| Song et al., 2017b | tomato | commercial organic fertilizer | 19670.70 | 1659.00 | 0.00 | 7.40 | 16.36 | 2 | Sand |
| Song et al., 2017b | tomato | commercial organic fertilizer | 14753.03 | 1244.25 | 0.00 | 7.40 | 16.36 | 2 | Sand |
| Song et al., 2017b | tomato | commercial organic fertilizer | 9835.35 | 829.50 | 0.00 | 7.40 | 16.36 | 2 | Sand |
| Song et al., 2017b | tomato | commercial organic fertilizer | 9835.35 | 829.50 | 273.33 | 7.40 | 16.36 | 2 | Sand |
| Wang et al., 2008 | sweet pepper | farmyard manure | 9700.50 | 352.50 | 0.00 | 6.75 | 14.09 | 1.164 | Loam |
| Wang et al., 2008 | sweet pepper | farmyard manure | 9700.50 | 352.50 | 143.49 | 6.75 | 14.09 | 1.164 | Loam |
| Wang et al., 2008 | sweet pepper | farmyard manure | 9700.50 | 352.50 | 286.99 | 6.75 | 14.09 | 1.164 | Loam |
| Kang et al., 2011 | tomato | farmyard manure |  | 450.00 | 0.00 |  | 7.34 | 0.84 | Sand |
| Kang et al., 2011 | tomato | farmyard manure |  | 270.00 | 180.00 |  | 7.34 | 0.84 | Sand |
| Kang et al., 2011 | tomato | farmyard manure |  | 180.00 | 270.00 |  | 7.34 | 0.84 | Sand |
| Kang et al., 2011 | tomato | farmyard manure |  | 270.00 | 0.00 |  | 7.34 | 0.84 | Sand |
| Xie et al., 2018 | tomato | farmyard manure | 601.36 | 418.02 | 0.00 | 6.50 | 21.11 |  | Loam |
| Xie et al., 2018 | tomato | farmyard manure | 498.94 | 346.82 | 163.46 | 6.50 | 21.11 |  | Loam |
| Xie et al., 2018 | tomato | farmyard manure | 345.27 | 240.00 | 394.23 | 6.50 | 21.11 |  | Loam |
| Xie et al., 2018 | tomato | farmyard manure | 191.60 | 133.18 | 625.00 | 6.50 | 21.11 |  | Loam |
| Xie et al., 2010 | tomato | farmyard manure | 12077.27 | 164.93 | 0.00 | 5.77 | 3.99 | 0.29 | Clay |
| Xie et al., 2010 | tomato | farmyard manure | 9663.24 | 131.96 | 101.50 | 5.77 | 3.99 | 0.29 | Clay |
| Xie et al., 2010 | tomato | farmyard manure | 7245.65 | 98.95 | 203.03 | 5.77 | 3.99 | 0.29 | Clay |
| Xie et al., 2010 | tomato | farmyard manure | 4739.32 | 64.72 | 304.53 | 5.77 | 3.99 | 0.29 | Clay |
| Hu, 2013 | cucumber | farmyard manure |  | 496.80 | 0.00 | 6.80 | 10.90 | 1.7 | Clay |
| Hu, 2013 | cucumber | farmyard manure |  | 714.00 | 0.00 | 6.80 | 10.90 | 1.7 | Clay |
| Hu, 2013 | cucumber | farmyard manure |  | 466.20 | 0.00 | 6.80 | 10.90 | 1.7 | Clay |
| Gao et al., 2017 | tomato | farmyard manure |  | 450.00 | 0.00 |  | 7.37 | 0.84 | Sand |
| Gao et al., 2017 | tomato | farmyard manure |  | 270.00 | 180.00 |  | 7.37 | 0.84 | Sand |
| Gao et al., 2017 | tomato | farmyard manure |  | 180.00 | 270.00 |  | 7.37 | 0.84 | Sand |
| Gao et al., 2017 | tomato | farmyard manure |  | 270.00 | 0.00 |  | 7.37 | 0.84 | Sand |
| Gao et al., 2017 | sweet pepper | farmyard manure |  | 450.00 | 0.00 |  | 7.37 | 0.84 | Sand |
| Gao et al., 2017 | sweet pepper | farmyard manure |  | 270.00 | 180.00 |  | 7.37 | 0.84 | Sand |
| Gao et al., 2017 | sweet pepper | farmyard manure |  | 180.00 | 270.00 |  | 7.37 | 0.84 | Sand |
| Gao et al., 2017 | sweet pepper | farmyard manure |  | 270.00 | 0.00 |  | 7.37 | 0.84 | Sand |
| Gao et al., 2017 | tomato | farmyard manure |  | 450.00 | 0.00 |  | 7.37 | 0.84 | Sand |
| Gao et al., 2017 | tomato | farmyard manure |  | 270.00 | 180.00 |  | 7.37 | 0.84 | Sand |
| Gao et al., 2017 | tomato | farmyard manure |  | 180.00 | 270.00 |  | 7.37 | 0.84 | Sand |
| Gao et al., 2017 | tomato | farmyard manure |  | 270.00 | 0.00 |  | 7.37 | 0.84 | Sand |
| Gao et al., 2017 | sweet pepper | farmyard manure |  | 450.00 | 0.00 |  | 7.37 | 0.84 | Sand |
| Gao et al., 2017 | sweet pepper | farmyard manure |  | 270.00 | 180.00 |  | 7.37 | 0.84 | Sand |
| Gao et al., 2017 | sweet pepper | farmyard manure |  | 180.00 | 270.00 |  | 7.37 | 0.84 | Sand |
| Gao et al., 2017 | sweet pepper | farmyard manure |  | 270.00 | 0.00 |  | 7.37 | 0.84 | Sand |
| Gao et al., 2017 | tomato | farmyard manure |  | 450.00 | 0.00 |  | 7.37 | 0.84 | Sand |
| Gao et al., 2017 | tomato | farmyard manure |  | 270.00 | 180.00 |  | 7.37 | 0.84 | Sand |
| Gao et al., 2017 | tomato | farmyard manure |  | 180.00 | 270.00 |  | 7.37 | 0.84 | Sand |
| Gao et al., 2017 | tomato | farmyard manure |  | 270.00 | 0.00 |  | 7.37 | 0.84 | Sand |
| Gao et al., 2017 | sweet pepper | farmyard manure |  | 450.00 | 0.00 |  | 7.37 | 0.84 | Sand |
| Gao et al., 2017 | sweet pepper | farmyard manure |  | 270.00 | 180.00 |  | 7.37 | 0.84 | Sand |
| Gao et al., 2017 | sweet pepper | farmyard manure |  | 180.00 | 270.00 |  | 7.37 | 0.84 | Sand |
| Gao et al., 2017 | sweet pepper | farmyard manure |  | 270.00 | 0.00 |  | 7.37 | 0.84 | Sand |
| Gao et al., 2017 | tomato | farmyard manure |  | 450.00 | 0.00 |  | 7.37 | 0.84 | Sand |
| Gao et al., 2017 | tomato | farmyard manure |  | 270.00 | 180.00 |  | 7.37 | 0.84 | Sand |
| Gao et al., 2017 | tomato | farmyard manure |  | 180.00 | 270.00 |  | 7.37 | 0.84 | Sand |
| Gao et al., 2017 | tomato | farmyard manure |  | 270.00 | 0.00 |  | 7.37 | 0.84 | Sand |
| Gao et al., 2017 | sweet pepper | farmyard manure |  | 450.00 | 0.00 |  | 7.37 | 0.84 | Sand |
| Gao et al., 2017 | sweet pepper | farmyard manure |  | 270.00 | 180.00 |  | 7.37 | 0.84 | Sand |
| Gao et al., 2017 | sweet pepper | farmyard manure |  | 180.00 | 270.00 |  | 7.37 | 0.84 | Sand |
| Gao et al., 2017 | sweet pepper | farmyard manure |  | 270.00 | 0.00 |  | 7.37 | 0.84 | Sand |
| Yang et al., 2006 | cucumber | compost | 9090.95 | 400.00 | 0.00 | 7.75 | 10.03 | 1.59 | Sand |
| Yang et al., 2006 | cucumber | farmyard manure | 14310.10 | 400.02 | 0.00 | 7.75 | 10.03 | 1.59 | Sand |
| Wang et al., 2016 | tomato | compost | 12127.50 | 588.00 | 0.00 | 7.47 | 18.44 | 1.77 | Sand |
| Wang et al., 2016 | tomato | compost | 24255.00 | 1176.00 | 0.00 | 7.47 | 18.44 | 1.77 | Sand |
| Wang et al., 2016 | tomato | compost | 48510.00 | 2352.00 | 0.00 | 7.47 | 18.44 | 1.77 | Sand |
| Wang et al., 2016 | tomato | compost | 5402.25 | 330.75 | 0.00 | 7.47 | 18.44 | 1.77 | Sand |
| Wang et al., 2016 | tomato | compost | 10804.50 | 661.50 | 0.00 | 7.47 | 18.44 | 1.77 | Sand |
| Wang et al., 2016 | tomato | compost | 21609.00 | 1323.00 | 0.00 | 7.47 | 18.44 | 1.77 | Sand |
| Wang et al., 2016 | heading cabbage | compost | 13702.50 | 651.00 | 0.00 | 7.47 | 18.44 | 1.77 | Sand |
| Wang et al., 2016 | heading cabbage | compost | 27405.00 | 1302.00 | 0.00 | 7.47 | 18.44 | 1.77 | Sand |
| Wang et al., 2016 | heading cabbage | compost | 54810.00 | 2604.00 | 0.00 | 7.47 | 18.44 | 1.77 | Sand |
| Wang et al., 2016 | tomato | compost | 6090.00 | 336.00 | 0.00 | 7.47 | 18.44 | 1.77 | Sand |
| Wang et al., 2016 | tomato | compost | 12180.00 | 672.00 | 0.00 | 7.47 | 18.44 | 1.77 | Sand |
| Wang et al., 2016 | tomato | compost | 24360.00 | 1344.00 | 0.00 | 7.47 | 18.44 | 1.77 | Sand |
| Wang et al., 2016 | heading cabbage | compost | 8079.75 | 309.75 | 0.00 | 7.47 | 18.44 | 1.77 | Sand |
| Wang et al., 2016 | heading cabbage | compost | 16159.50 | 619.50 | 0.00 | 7.47 | 18.44 | 1.77 | Sand |
| Wang et al., 2016 | heading cabbage | compost | 32319.00 | 1239.00 | 0.00 | 7.47 | 18.44 | 1.77 | Sand |
| Chen et al., 2020 | cabbage | compost |  | 100.10 | 150.15 | 8.43 | 6.09 |  | Sand |
| Chen et al., 2020 | cabbage | compost |  | 255.27 | 0.00 | 8.43 | 6.09 |  | Sand |
| Chen et al., 2020 | pepper | compost |  | 205.02 | 307.53 | 8.43 | 6.09 |  | Sand |
| Chen et al., 2020 | pepper | compost |  | 500.49 | 0.00 | 8.43 | 6.09 |  | Sand |
| Chen et al., 2020 | cabbage | compost |  | 145.12 | 217.68 | 8.43 | 6.09 |  | Sand |
| Chen et al., 2020 | cabbage | compost |  | 363.81 | 0.00 | 8.43 | 6.09 |  | Sand |
| Chen et al., 2020 | eggplant | compost |  | 160.20 | 240.30 | 8.43 | 6.09 |  | Sand |
| Chen et al., 2020 | eggplant | compost |  | 406.02 | 0.00 | 8.43 | 6.09 |  | Sand |
| Chen et al., 2020 | cherry tomato | compost |  | 185.12 | 277.68 | 8.43 | 6.09 |  | Sand |
| Chen et al., 2020 | cherry tomato | compost |  | 462.30 | 0.00 | 8.43 | 6.09 |  | Sand |
| Chen et al., 2020 | eggplant | compost |  | 160.20 | 240.30 | 8.43 | 6.09 |  | Sand |
| Chen et al., 2020 | eggplant | compost |  | 402.00 | 0.00 | 8.43 | 6.09 |  | Sand |
| Chen et al., 2020 | cherry tomato | compost |  | 185.12 | 277.68 | 8.43 | 6.09 |  | Sand |
| Chen et al., 2020 | cherry tomato | compost |  | 464.31 | 0.00 | 8.43 | 6.09 |  | Sand |
| Chen et al., 2020 | tomato | compost |  | 190.15 | 285.22 | 8.43 | 6.09 |  | Sand |
| Chen et al., 2020 | tomato | compost |  | 468.33 | 0.00 | 8.43 | 6.09 |  | Sand |
| Chen et al., 2020 | fennel | compost |  | 201.00 | 301.50 | 8.43 | 6.09 |  | Sand |
| Chen et al., 2020 | fennel | compost |  | 387.93 | 0.00 | 8.43 | 6.09 |  | Sand |
| Chen et al., 2020 | eggplant | compost |  | 160.20 | 240.30 | 8.43 | 6.09 |  | Sand |
| Chen et al., 2020 | eggplant | compost |  | 395.97 | 0.00 | 8.43 | 6.09 |  | Sand |
| Chang et al., 2007 | pakchoi | farmyard manure | 3501.45 | 442.43 | 0.00 | 7.54 | 14.96 | 1.62 | Loam |
| Chang et al., 2007 | pakchoi | farmyard manure | 5102.81 | 439.05 | 0.00 | 7.54 | 14.96 | 1.62 | Loam |
| Chang et al., 2007 | pakchoi | compost | 10812.87 | 615.52 | 0.00 | 7.54 | 14.96 | 1.62 | Loam |
| Chang et al., 2007 | pakchoi | compost | 9187.02 | 736.26 | 0.00 | 7.54 | 14.96 | 1.62 | Loam |
| Chang et al., 2007 | lettuce | farmyard manure | 3501.45 | 442.43 | 0.00 | 7.54 | 14.96 | 1.62 | Loam |
| Chang et al., 2007 | lettuce | farmyard manure | 5102.81 | 439.05 | 0.00 | 7.54 | 14.96 | 1.62 | Loam |
| Chang et al., 2007 | lettuce | compost | 10812.87 | 615.52 | 0.00 | 7.54 | 14.96 | 1.62 | Loam |
| Chang et al., 2007 | lettuce | compost | 9187.02 | 736.26 | 0.00 | 7.54 | 14.96 | 1.62 | Loam |
| Chang et al., 2007 | amaranth | farmyard manure | 3501.45 | 442.43 | 0.00 | 7.54 | 14.96 | 1.62 | Loam |
| Chang et al., 2007 | amaranth | farmyard manure | 5102.81 | 439.05 | 0.00 | 7.54 | 14.96 | 1.62 | Loam |
| Chang et al., 2007 | amaranth | compost | 10812.87 | 615.52 | 0.00 | 7.54 | 14.96 | 1.62 | Loam |
| Chang et al., 2007 | amaranth | compost | 9187.02 | 736.26 | 0.00 | 7.54 | 14.96 | 1.62 | Loam |
| Chang et al., 2007 | amaranth | farmyard manure | 3501.45 | 442.43 | 0.00 | 7.54 | 14.96 | 1.62 | Loam |
| Chang et al., 2007 | amaranth | farmyard manure | 5102.81 | 439.05 | 0.00 | 7.54 | 14.96 | 1.62 | Loam |
| Chang et al., 2007 | amaranth | compost | 10812.87 | 615.52 | 0.00 | 7.54 | 14.96 | 1.62 | Loam |
| Chang et al., 2007 | amaranth | compost | 9187.02 | 736.26 | 0.00 | 7.54 | 14.96 | 1.62 | Loam |
| Li et al., 2017b | tomato | compost | 5275.04 | 350.00 | 0.00 | 8.07 | 11.02 | 1.34 | Loam |
| Li et al., 2017b | tomato | farmyard manure | 7172.67 | 350.00 | 0.00 | 8.07 | 11.02 | 1.34 | Loam |
| Li et al., 2017b | tomato | compost | 4060.00 | 350.00 | 0.00 | 8.07 | 11.02 | 1.34 | Loam |
| Li et al., 2017b | celery | compost | 5275.04 | 350.00 | 0.00 | 8.07 | 11.02 | 1.34 | Loam |
| Li et al., 2017b | celery | farmyard manure | 7172.67 | 350.00 | 0.00 | 8.07 | 11.02 | 1.34 | Loam |
| Li et al., 2017b | celery | compost | 4060.00 | 350.00 | 0.00 | 8.07 | 11.02 | 1.34 | Loam |
| Li et al., 2017b | tomato | compost | 5275.04 | 350.00 | 0.00 | 8.07 | 11.02 | 1.34 | Loam |
| Li et al., 2017b | tomato | farmyard manure | 7172.67 | 350.00 | 0.00 | 8.07 | 11.02 | 1.34 | Loam |
| Li et al., 2017b | tomato | compost | 4060.00 | 350.00 | 0.00 | 8.07 | 11.02 | 1.34 | Loam |
| Zhang et al., 2016 | amaranth | compost | 976.40 | 312.60 | 0.00 | 5.52 | 9.05 | 1.9 | Loam |
| Zhang et al., 2016 | baby bok choy | compost | 375.76 | 120.30 | 0.00 | 5.52 | 9.05 | 1.9 | Loam |
| Zhang et al., 2016 | coriander herb | compost | 976.40 | 312.60 | 0.00 | 5.52 | 9.05 | 1.9 | Loam |
| Zhang et al., 2016 | amaranth | compost | 976.40 | 312.60 | 0.00 | 5.52 | 9.05 | 1.9 | Loam |
| Zhang et al., 2016 | tung choy | compost | 897.69 | 287.40 | 0.00 | 5.52 | 9.05 | 1.9 | Loam |
| Zhang et al., 2016 | baby bok choy | compost | 780.56 | 249.90 | 0.00 | 5.52 | 9.05 | 1.9 | Loam |
| Zhang et al., 2016 | coriander herb | compost | 976.40 | 312.60 | 0.00 | 5.52 | 9.05 | 1.9 | Loam |
| Zhang et al., 2016 | tung choy | compost | 976.40 | 312.60 | 0.00 | 5.52 | 9.05 | 1.9 | Loam |
| Zhang et al., 2016 | baby bok choy | compost | 780.56 | 249.90 | 0.00 | 5.52 | 9.05 | 1.9 | Loam |
| Zhang et al., 2016 | amaranth | compost | 780.56 | 249.90 | 0.00 | 5.52 | 9.05 | 1.9 | Loam |
| Song et al., 2017a | cucumber | commercial organic fertilizer |  | 465.00 | 0.00 | 7.46 | 13.15 | 1.85 | Loam |
| Song et al., 2017a | cucumber | commercial organic fertilizer |  | 465.00 | 0.00 | 7.46 | 13.15 | 1.85 | Loam |
| Song et al., 2017a | cucumber | commercial organic fertilizer |  | 465.00 | 0.00 | 7.46 | 13.15 | 1.85 | Loam |
| Song et al., 2017a | cucumber | commercial organic fertilizer |  | 465.00 | 0.00 | 7.46 | 13.15 | 1.85 | Loam |
| Song et al., 2017a | purple cabbage | commercial organic fertilizer |  | 232.50 | 0.00 | 7.46 | 13.15 | 1.85 | Loam |
| Song et al., 2017a | purple cabbage | commercial organic fertilizer |  | 232.50 | 0.00 | 7.46 | 13.15 | 1.85 | Loam |
| Song et al., 2017a | purple cabbage | commercial organic fertilizer |  | 232.50 | 0.00 | 7.46 | 13.15 | 1.85 | Loam |
| Song et al., 2017a | purple cabbage | commercial organic fertilizer |  | 232.50 | 0.00 | 7.46 | 13.15 | 1.85 | Loam |
| Li et al., 2018 | cucumber | farmyard manure |  | 918.00 | 1590.00 | 8.30 | 18.80 | 1.3 | Sand |
| Li et al., 2018 | cucumber | farmyard manure |  | 918.00 | 1246.00 | 8.30 | 18.80 | 1.3 | Sand |
| Li et al., 2018 | tomato-cucumber | farmyard manure |  | 362.00 | 1877.00 | 8.00 | 20.70 | 2.1 | Sand |
| Li et al., 2018 | tomato-cucumber | farmyard manure |  | 362.00 | 1354.00 | 8.00 | 20.70 | 2.1 | Sand |
| Wen et al., 2013 | celery | commercial organic fertilizer | 126.35 | 99.93 | 0.00 | 7.20 | 10.73 |  | Sand |
| Wen et al., 2013 | celery | commercial organic fertilizer | 252.70 | 199.87 | 0.00 | 7.20 | 10.73 |  | Sand |
| Wen et al., 2013 | celery | commercial organic fertilizer | 505.40 | 399.74 | 0.00 | 7.20 | 10.73 |  | Sand |
| Wen et al., 2013 | celery | commercial organic fertilizer | 1010.80 | 799.47 | 0.00 | 7.20 | 10.73 |  | Sand |
| Wen et al., 2013 | celery | commercial organic fertilizer | 2021.60 | 1598.94 | 0.00 | 7.20 | 10.73 |  | Sand |
| Wen et al., 2013 | celery | commercial organic fertilizer | 4043.20 | 3197.88 | 0.00 | 7.20 | 10.73 |  | Sand |
| Wen et al., 2013 | tomato | commercial organic fertilizer | 126.35 | 99.93 | 0.00 | 7.20 | 10.73 |  | Sand |
| Wen et al., 2013 | tomato | commercial organic fertilizer | 252.70 | 199.87 | 0.00 | 7.20 | 10.73 |  | Sand |
| Wen et al., 2013 | tomato | commercial organic fertilizer | 505.40 | 399.74 | 0.00 | 7.20 | 10.73 |  | Sand |
| Wen et al., 2013 | tomato | commercial organic fertilizer | 1010.80 | 799.47 | 0.00 | 7.20 | 10.73 |  | Sand |
| Wen et al., 2013 | tomato | commercial organic fertilizer | 2021.60 | 1598.94 | 0.00 | 7.20 | 10.73 |  | Sand |
| Wen et al., 2013 | tomato | commercial organic fertilizer | 4043.20 | 3197.88 | 0.00 | 7.20 | 10.73 |  | Sand |
| Wen et al., 2013 | chrysanthemum | commercial organic fertilizer | 126.35 | 99.93 | 0.00 | 7.20 | 10.73 |  | Sand |
| Wen et al., 2013 | chrysanthemum | commercial organic fertilizer | 252.70 | 199.87 | 0.00 | 7.20 | 10.73 |  | Sand |
| Wen et al., 2013 | chrysanthemum | commercial organic fertilizer | 505.40 | 399.74 | 0.00 | 7.20 | 10.73 |  | Sand |
| Wen et al., 2013 | chrysanthemum | commercial organic fertilizer | 1010.80 | 799.47 | 0.00 | 7.20 | 10.73 |  | Sand |
| Wen et al., 2013 | chrysanthemum | commercial organic fertilizer | 2021.60 | 1598.94 | 0.00 | 7.20 | 10.73 |  | Sand |
| Wen et al., 2013 | chrysanthemum | commercial organic fertilizer | 4043.20 | 3197.88 | 0.00 | 7.20 | 10.73 |  | Sand |
| Wang et al., 2017a | tomato | commercial organic fertilizer | 1991.52 | 161.10 | 90.00 | 6.33 | 25.71 |  | Loam |
| Li et al., 2017a | tomato | commercial organic fertilizer |  | 32.67 | 115.31 | 8.22 |  |  | Loam |
| Zhang et al., 2019b | tomato | commercial organic fertilizer |  | 190.29 | 0.00 | 7.20 | 7.73 |  | Loam |
| Zhang et al., 2019b | tomato | commercial organic fertilizer |  | 190.29 | 393.00 | 7.20 | 7.73 |  | Loam |
| Zhang et al., 2019b | tomato | commercial organic fertilizer |  | 247.40 | 393.00 | 7.20 | 7.73 |  | Loam |
| Tang, 2009 | bok choy | compost | 5110.00 | 420.00 | 0.00 | 7.75 | 1.35 | 14.7 | Sand |
| Tang, 2009 | bok choy | compost | 10220.00 | 850.00 | 0.00 | 7.75 | 1.35 | 14.7 | Sand |
| Tang, 2009 | tomato | compost | 6390.00 | 360.00 | 0.00 | 7.75 | 1.35 | 14.7 | Sand |
| Tang, 2009 | tomato | compost | 12780.00 | 710.00 | 0.00 | 7.75 | 1.35 | 14.7 | Sand |
| Tang, 2009 | cucumber | compost | 3300.00 | 260.00 | 0.00 | 7.75 | 1.35 | 14.7 | Sand |
| Tang, 2009 | cucumber | compost | 6600.00 | 520.00 | 0.00 | 7.75 | 1.35 | 14.7 | Sand |
| Song, 2008 | tomato | compost | 23229.00 | 1372.50 | 0.00 | 7.57 | 10.99 | 1.34 | Loam |
| Song, 2008 | tomato | compost | 20749.50 | 1530.00 | 0.00 | 7.57 | 10.99 | 1.34 | Loam |
| Song, 2008 | tomato | farmyard manure | 5272.20 | 452.25 | 0.00 | 7.57 | 10.99 | 1.34 | Loam |
| Song, 2008 | tomato | farmyard manure | 10544.40 | 904.50 | 0.00 | 7.57 | 10.99 | 1.34 | Loam |
| Song, 2008 | tomato | farmyard manure | 15816.60 | 1356.75 | 0.00 | 7.57 | 10.99 | 1.34 | Loam |
| Song, 2008 | celery | compost | 23229.00 | 1372.50 | 0.00 | 7.57 | 10.99 | 1.34 | Loam |
| Song, 2008 | celery | compost | 20749.50 | 1530.00 | 0.00 | 7.57 | 10.99 | 1.34 | Loam |
| Song, 2008 | celery | farmyard manure | 5272.20 | 452.25 | 0.00 | 7.57 | 10.99 | 1.34 | Loam |
| Song, 2008 | celery | farmyard manure | 10544.40 | 904.50 | 0.00 | 7.57 | 10.99 | 1.34 | Loam |
| Song, 2008 | celery | farmyard manure | 15816.60 | 1356.75 | 0.00 | 7.57 | 10.99 | 1.34 | Loam |
| Song, 2008 | tomato | compost | 23229.00 | 1372.50 | 0.00 | 7.57 | 10.99 | 1.34 | Loam |
| Song, 2008 | tomato | compost | 20749.50 | 1530.00 | 0.00 | 7.57 | 10.99 | 1.34 | Loam |
| Song, 2008 | tomato | farmyard manure | 5272.20 | 452.25 | 0.00 | 7.57 | 10.99 | 1.34 | Loam |
| Song, 2008 | tomato | farmyard manure | 10544.40 | 904.50 | 0.00 | 7.57 | 10.99 | 1.34 | Loam |
| Song, 2008 | tomato | farmyard manure | 15816.60 | 1356.75 | 0.00 | 7.57 | 10.99 | 1.34 | Loam |
| Wang et al., 2017b | Cucumber | commercial organic fertilizer |  | 400.00 | 0.00 | 5.08 | 26.80 | 2.32 | Loam |
| Zheng et al., 2018 | Cucumber | farmyard manure | 443.02 | 22.77 | 90.00 | 7.80 | 12.93 | 1.93 | Loam |
| Zheng et al., 2018 | Cucumber | farmyard manure | 886.04 | 45.54 | 90.00 | 7.80 | 12.93 | 1.93 | Loam |
| Zheng et al., 2018 | Cucumber | farmyard manure | 3544.17 | 182.16 | 90.00 | 7.80 | 12.93 | 1.93 | Loam |
| Zhao et al., 2012 | Tomato | farmyard manure | 3200.40 | 172.80 | 750.00 | 8.27 | 17.46 | 1.96 | Loam |
| Zhao et al., 2012 | Cucumber | farmyard manure | 3434.40 | 165.60 | 675.00 | 8.27 | 17.46 | 1.96 | Loam |
| Zhao et al., 2012 | Tomato | farmyard manure | 2856.60 | 81.00 | 525.00 | 8.27 | 17.46 | 1.96 | Loam |
| Zhao et al., 2012 | Cucumber | farmyard manure | 2122.20 | 81.00 | 450.00 | 8.27 | 17.46 | 1.96 | Loam |
| Quan et al., 2015 | cucumber | farmyard manure | 4700.00 | 370.00 | 0.00 | 6.72 | 13.00 | 1.13 | Loam |
| Quan et al., 2015 | cucumber | farmyard manure | 7050.00 | 555.00 | 0.00 | 6.72 | 13.00 | 1.13 | Loam |
| Zhu et al., 2005 | pepper | farmyard manure |  | 178.00 | 0.00 | 6.61 | 10.09 | 3 | Sand |
| Zhu et al., 2005 | pepper | farmyard manure |  | 178.00 | 600.00 | 6.61 | 10.09 | 3 | Sand |
| Zhu et al., 2005 | pepper | farmyard manure |  | 178.00 | 1200.00 | 6.61 | 10.09 | 3 | Sand |
| Zhu et al., 2005 | pepper | farmyard manure |  | 178.00 | 1800.00 | 6.61 | 10.09 | 3 | Sand |
| Xu et al., 2020 | pepper | compost | 4000.00 | 322.77 | 180.00 | 7.82 | 16.30 | 0.97 | Loam |
| Xu et al., 2020 | pepper | compost | 4000.00 | 265.74 | 180.00 | 7.82 | 16.30 | 0.97 | Loam |
| Xu et al., 2020 | pepper | compost | 4000.00 | 408.96 | 180.00 | 7.82 | 16.30 | 0.97 | Loam |
| Xu et al., 2020 | pepper | compost | 4000.00 | 453.03 | 180.00 | 7.82 | 16.30 | 0.97 | Loam |
| Xu et al., 2020 | pepper | compost | 4000.00 | 297.75 | 180.00 | 7.82 | 16.30 | 0.97 | Loam |
| Xia and Yang, 2003 | tomato | farmyard manure | 2583.00 | 201.08 | 255.00 |  | 10.58 | 1.26 | Loam |
| Xia and Yang, 2003 | tomato | farmyard manure | 5166.00 | 402.15 | 600.00 |  | 10.58 | 1.26 | Loam |
| Xia and Yang, 2003 | tomato | farmyard manure | 5166.00 | 402.15 | 255.00 |  | 10.58 | 1.26 | Loam |
| Xia and Yang, 2003 | tomato | farmyard manure | 2583.00 | 201.08 | 600.00 |  | 10.58 | 1.26 | Loam |
| Xia and Yang, 2003 | tomato | farmyard manure | 2583.00 | 201.08 | 0.00 |  | 10.58 | 1.26 | Loam |
| Xia and Yang, 2003 | tomato | farmyard manure | 5166.00 | 402.15 | 0.00 |  | 10.58 | 1.26 | Loam |

**Note:**

**Initial SOC** **:** initial SOC content**；initial STN:** initial STN content.

**Notes S1. Reference list for the meta-analysis**

Cai, R., Xiao, Y., Wu, X., Wang, X., Xu, X., 2019. Effects of frass manure on soil physical and chemical properties and tomato yield in greenhouse. Journal of Henan Agricultural Sciences 48, 70-74. (in Chinese with English abstract).

Chang, E., Chung, R., Tsai, Y., 2007. Effect of different application rates of organic fertilizer on soil enzyme activity and microbial population. Soil Science and Plant Nutrition 53, 132-140.

Chen, S., Yan, Z., Ha, X., Qin, W., Chen, Q., 2020. Combining application of chemical fertilizer with manure significantly increased potassium availability in an alkaline soil. Nutr Cycl Agroecosyst, https://doi.org/10.1007/s10705-10019-10044-x.

Duan, P., 2016. Effect of combined application n fertilizer and manure on soil n supply capacity in greenhouse condition. Shenyang Agriculture University, Shenyang. (in Chinese with English abstract).

Feng, H., Zhang, L., Qu, J., Yang, D., Guo, W., Li, Q., 2015. Effects of soil nutrients,microbial and yield of celery in facilities soil under green manure and fertilizer reduction. Journal of Gansu Agricultural University 50, 66-70. (in Chinese with English abstract).

Gao, H., Ge, X., 2005. Effects of different fertilizer application on dry matter distribution and yield and quality of tomato in greenhouse. Northern Horticulture, 38-40. (in Chinese with English abstract).

Gao, J., Kang, L., Yan, Z., Qu, M., Liu, Z., Cuizhen, Z., Chen, Qing, 2017. Effects of biogas manure replacing chemical fertilizer on accumulation of nutrient and heavy metal in greenhouse vegetable soil. Transactions of the Chinese Society of Agricultural Engineering 33, 200-207. (in Chinese with English abstract).

Gao, X., 2019. Effects of integrated application of biogas slurry water and fertilizer instead of chemical fertilizer on greenhouse crops and soil properties. Northwest A&F University, Yangling. (in Chinese with English abstract).

Ge, X.g., Gao, H., Zhang, E., Wang, X., Zhang, X., 2004. Studies on changes of field - vegetable system under long-term fixed fertilizer experiment(ⅲ) changes of vegetable yield and nutrient absorption under different fertilization systems. Acta Horticulturae Sinica 31, 456-460. (in Chinese with English abstract).

Han, D., 2016. The Effects of combined phosphate fertilizer and organic manure on tomato yield,quality and accumulation of heavy metals. Shenyang Agriculture University, Shenyang. (in Chinese with English abstract).

Hu, X., 2015. Effects of green manures on soil properties and afterreap eggplant output and quality in greenhouse. Hainan University, Haikou. (in Chinese with English abstract).

Hu, Y., 2013. Effect of biogas manure on agronomic traits and yield of cucumber in plastic greenhouse. Northern Horticulture, 154-156. (in Chinese with English abstract).

Huang, D., 2016. Effects of different materials on the growth and development of tomato in greenhouse. Shenyang Agriculture University, Shenyang. (in Chinese with English abstract).

Kang, L., Zhao, Y., Qu, M., Chen, Q., 2011. Effects of biogas waste on solanaceae vegetable growth and soil nutrient accumulation in greenhouse. CHINA VEGETABLES, 57-62. (in Chinese with English abstract).

Li, C., 2012. The research on decompostion properties of different organic fertilizer and effect of increasing soil fertility. Guangxi University, Nanning. (in Chinese with English abstract).

Li, J., Liu, H., Wang, H., Luo, J., Zhang, X., Liu, Z., Zhang, Y., Zhai, L., Lei, Q., Ren, T., Li, Y., Bashir, M., 2018. Managing irrigation and fertilization for the sustainable cultivation of greenhouse vegetables. Agricultural Water Management 210, 354-363.

Li, M., Nie, H., Zhang, S., Zhou, F., Han, D., Zhan, L., Tian, Y., Shi, M., Zhang, E., 2016. Correlation between fda enzyme activity and soil fertility under combining application organic and nitrogen fertilizer in facility vegetable field. Acta Horticulturae Sinica 43, 907-917. (in Chinese with English abstract).

Li, R., Chen, W., Cai, F., Zhao, Z., Gao, R., Long, X., 2017a. Effects of Trichoderma-enriched biofertilizer on tomato plant growth and fruit quality. Journal of Nanjing Agricultural University 40, 464-472. (in Chinese with English abstract).

Li, S., Li, J., Zhang, B., Li, D., Li, G., Li, Y., 2017b. Effect of different organic fertilizers application on growth and environmental risk of nitrate under a vegetable field. Scientific Reports 7, 17020.

Li, W., 2004. The study of altering regularity about the nitrate content of the fruit and greenhouse soil of mini cucumber and cherry tomato in the different conditions of applying fertilizer. Ningxia University, Yinchuan. (in Chinese with English abstract).

Li, Z., He, H., Wang, W., Li, H., Zhao, T., Zhou, X., 2015. Effect of different organic fertilizer on greenhouse cucumber and soil. Northern Horticulture, 178-181. (in Chinese with English abstract).

Liu, L., Feng, N., Chen, J., Li, H., 2014. Effects of organic manure/chemical fertilizer ratio on pakchoi yield and quality in greenhouse and open field under the same N fertilizer rate. Acta Agriculturae Shanghai 30, 29-33. (in Chinese with English abstract).

Liu, Z., Wu, X., Li, R., Zheng, F., Zhang, M., Li, S., Song, X., 2019. Effect of applying chicken manure and phosphate fertilizer on soil phosphorus under drip irrigation in greenhouse. 52, 3637-3647. (in Chinese with English abstract).

Luan, H., Gao, W., Huang, S., Tang, J., Li, M., Zhang, H., Chen, X., Masiliūnas, D., 2020. Substitution of manure for chemical fertilizer affects soil microbial community diversity, structure and function in greenhouse vegetable production systems. PLOS ONE 15, e0214041.

Luo, J., Liu, L., Wang, T., Liu, H., Yan, S., Lu, X., Fan, R., Zhang, Z., 2015. Effect of organic fertilizer from deep-litter pig rearing on pepper yield and soil microbial diversity. Soils 47, 1101–1106. (in Chinese with English abstract).

Luo, J., Liu, L., Wang, T., Yan, S., Lu, X., Fan, R., Zhang, Z., 2016. Effect of combined application of chemical fertilizer with organic manure on cucumber yield and soil microbial diversity. Journal of Ecology and Ｒural Environment 32, 774-779. (in Chinese with English abstract).

Lv, N., Shen, Z., Wang, D., Liu, H., Xue, C., Li, R., Shen, Q., 2018. Effects of amino acid organic fertilizer on cucumber yield and soil biological characters. Journal of Nanjing Agricultural University 41, 456-464. (in Chinese with English abstract).

Quan, Z., Lu, C., Shi, Y., Chen, X., Huang, B., Wang, Y., Zhao, Y., Ma, J., 2015. Manure increase the leaching risk of soil extractable organic nitrogen in intensively irrigated greenhouse vegetable cropping systems. Acta Agriculturae Scandinavica, Section B — Soil & Plant Science 65, 199-207.

Ren, R., 2018. Effect of different fertilization methods on soil potassium availability and yield and quality of tomato. Shenyang Agriculture University, Shenyang. (in Chinese with English abstract).

Shen, L., Bai, L., Zeng, X., Wang, Y., 2012. Effects of Fertilization on NO^-3^-N Accumulation in Greenhouse Soils. Journal of Agro-Environment Science 31, 1350-1356. (in Chinese with English abstract).

Song, D., 2008. Transformation of manure in soil and effect on the growth of organic vegetable. Shangdong Agriculture University, Taian. (in Chinese with English abstract).

Song, K., Xue, Y., Zheng, X., Lv, W., Qiao, H., Qin, Q., Yang, J., 2017a. Effects of the continuous use of organic manure and chemical fertilizer on soil inorganic phosphorus fractions in calcareous soil. Scientific reports 7, 1164.

Song, Y., Ma, M., An, Z., Zhao, T., 2017b. Effect of combined application of organic and inorganic fertilizers on the yield and quality of pepper and tomato in greenhouse. Acta Agriculturae Boreali-Sinica 32, 211-216. (in Chinese with English abstract).

Tan, F., 2016. Effects of long-term located fertilization on soil physicochemical properties,micro organism and yield and quality of tomato. Shenyang Agriculture University,, Shenyang. (in Chinese with English abstract).

Tang, Z., 2009. Studies on agronomic and environmental effects by different water and fertilizer managements in organic vegetable farming. Chinese Academy of Agricultural Sciences, Beijing. (in Chinese with English abstract).

Tian, Y., 2018. Effects of long-term potassium deficiency on tomato root exudates and rhizosphere microecology. Shenyang Agriculture University, Shenyang. (in Chinese with English abstract).

Wan, S., Zhang, S., Zhao, M., Sun, Y., Zhao, Z., Cai, K., Wang, W., 2013. Effect of soybean meal combined application of chemical fertilizer on greenhouse cucumber yield and quality and soil fertility. Chinese Agricultural Science Bulletin 29, 188-193. (in Chinese with English abstract).

WANG, D., Chen, L., Huang, Z., Sun, F., Diao, C., Xu, M., Wu, X., Liu, Q., Wu, Z., Li, W., 2017a. Effects of fermentation residues of CO_2_ fertilization on tomato yield and quality. Soils 49, 893–896. (in Chinese with English abstract).

Wang, F., Li, G., Wang, L., Zhou, J., Lou, Y., Yao, H., 2017b. Examining the effects of reduced nitrogen fertilization on the yield and fruit quality of greenhouse cultivated cucumber. Chinese Journal of Applied Ecology 28, 3627-3633. (in Chinese with English abstract).

Wang, M., Yan, Z., Chen, S., Gao, J., Li, J., Xu, J., Chen, Q., 2016. Effects of manure and biogas slurry applications on phosphorus accumulation and mobility in organic vegetable soil under greenhouse. Journal of Agro-Environment Science 35, 1351-1359. (in Chinese with English abstract).

Wang, X., 2017. Effect of combined application n fertilizer and manure on soil respiration in greenhouse condition. Shenyang Agriculture University, Shenyang. (in Chinese with English abstract).

Wang, Y., Zhang, E., Zhang, S., Zhao, H., 2008. Effects of long-term application of manure and nitrogen on soil fertility and sweet pepper yield. Northern Horticulture, 63-65. (in Chinese with English abstract).

Wen, F., Han, B., Jin, Q., Li, S., Liang, J., Li, P., 2013. A study on the effect of commercial organic fertilizer application on soil fertility and crop growth in newly-built facilities. China Agricultural Technology Extension 29, 42-44. (in Chinese with English abstract).

Wu, R., Liu, S., Sun, H., Tian, Y., Chen, Y., Du, Y., Ma, L., 2018. Study on the effect of organic nitrogen and inorganic nitrogen on the improvement of secondary salinized soil. Soil and Fertilizer Sciences in China, 61-66. (in Chinese with English abstract).

Xia, L., Yang, L., 2003. Effect of fertilizer application on the accumulation of soil nutrients in plastic tunnel for tomato cultivation. CHINA VEGETABLES, 4-7. (in Chinese with English abstract).

Xie, H., Dong, R., Wu, S., Yang, S., Yang, J., 2018. Effect of biogas slurry combined with chemical fertilizer on the yield and quality of tomato growth in greenhouse. Soil and Fertilizer Sciences in China, 108-115. (in Chinese with English abstract).

Xie, J., Chen, G., Yuan, Q., Lin, G., Wang, Z., Guo, C., Zhong, H., 2010. Effects of combined application of biogas residues and chemical fertilizers on greenhouse tomato’s growth and its fruit yield and quality. Chinese Journal of Applied Ecology 21, 2353-2357. (in Chinese with English abstract).

Xu, F., Liang, Y., Zhang, C., Du, S., Chen, Z., 2004. Effect of fertilization on cucumber growth and soil biological characteristics in sunlight greenhouse. Chinese Journal of Applied Ecology 15, 1227-1230. (in Chinese with English abstract).

Xu, Y., Ma, Y., Cayuela, M.L., Sánchez-Monedero, M.A., Wang, Q., 2020. Compost biochemical quality mediates nitrogen leaching loss in a greenhouse soil under vegetable cultivation. Geoderma 358, 113984.

Yang, H., Fan, J., Niu, X., Li, J., 2006. Study on the effects of application of fen manure and biological-organic-inorganic compound fertilizer to vegetable in greenhouse. Acta Agriculturae Boreali-Sinica 21, 63-67. (in Chinese with English abstract).

Yang, L., Yang, Q., Zhou, C., Chang, Q., 2013. Effect of vermicompost on tomato yield and quality and soil microorganisms population in greenhouse. Chinese Journal of Soil Science 44, 1455-1459. (in Chinese with English abstract).

Yao, X., 2012. Effect of different kinds of organic fertilizers on the fruit yeild quality and soil respiration characteristics of pepper. Northwest A&F University, Yangling. (in Chinese with English abstract).

Zhang, G., 2004. ffect of fertilization levels on growth development of tomato and siol environment in helio-greenhouse. China Agriculture University, Beijing. (in Chinese with English abstract).

Zhang, G., Bai, Y., Fan, Y., Yang, Q., Yang, L., 2019a. Effect of organic materials combined application with chemical fertilizer on accumulation,distribution and utilization efficiency of nitrogen in greenhouse tomato. Northern Horticulture, 6-13. (in Chinese with English abstract).

Zhang, G., Fan, Y., Zhao, F., Zhang, Y., Yang, Q., Yang, L., 2018. Effects of organic materials combined application with mineral fertilizer on the growth,photosynthesis,yield and quality of tomato in greenhouse. China Sciencepaper 13, 698-703. (in Chinese with English abstract).

Zhang, J., QU, F., Zhu, Y., Yang, J., Hu, X., 2019b. Effects of more organic fertilizer and microbial agents on yield and quality of spring greenhouse tomato in Yangling. Acta Agriculturae Boreali-occidentalis Sinica 28, 767-773. (in Chinese with English abstract).

Zhang, J., Zhou, F., 2012. Effects of different ratio of rape-seed cakes and pig manure application together on the plant growth,yield and quality of greenhouse cherry tomato. Guangdong Agricultural Sciences, 90-30. (in Chinese with English abstract).

Zhang, M., Li, B., Xiong, Z.Q., 2016. Effects of organic fertilizer on net global warming potential under an intensively managed vegetable field in southeastern China: A three-year field study. Atmospheric Environment 145, 92-103.

Zhang, Y., 2018. Effects of straw and/or lime addition on soil aggregate composition,tomato yield and quality in greenhouse. Shenyang Agriculture University, Shenyang. (in Chinese with English abstract).

Zhang, Y., Liu, H., Zhao, F., Zhang, G., Li, M., Yang, L., 2017. Regulation of various fertilization measures on soil microbial functional diversity and phenolic acid contents under tomato continuous cropping in greenhouse. Chinese Journal of Soil Science 48, 887- 894. (in Chinese with English abstract).

Zhao, M., Cai, K., Sun, Y., Zhao, Z., Zhu, Z., 2014. Carbon,nitrogen mineralization and effects on yield and quality of tomato in greenhouse of biochar made by sludge. Chinese Agricultural Science Bulletin 30, 215-220. (in Chinese with English abstract).

Zhao, M., Wang, W., Cai, k., Zhao, Z., Chen, J., 2009. Effects of combining application of organic and inorganic fertilizers on cucumber quality and yield in greenhouse. Northern Horticulture, 137-140. (in Chinese with English abstract).

Zhao, Y., Li, Y., Dong, H., Ji, P., Shi, Y., Xu, H., Wu, L., Feng, W., 2010. Effects of manure application methods on growth of greenhouse vegetables and soil fertility. Jiangsu Agricultural Sciences, 187-189. (in Chinese with English abstract).

Zhao, Y., Luo, J.-H., Chen, X.-Q., Zhang, X.-J., Zhang, W.-L., 2012. Greenhouse tomato–cucumber yield and soil N leaching as affected by reducing N rate and adding manure: a case study in the Yellow River Irrigation Region China. Nutrient Cycling in Agroecosystems 94, 221-235.

Zheng, S., Zhao, H., Wu, Y., Zhao, L., Li, T., Qian, R., Shan, Y., Feng, K., 2018. Effects of vermicompost instead of partial inorganic base fertilizer on the growth of continuous cropping cucumber and soil properties in greenhouse. Acta Agriculturae Shanghai 34, 1-7. (in Chinese with English abstract).

Zhou, D., Yang, L., 2013. Effect of different fertilizer combinations on yield and quality of tomato in greenhouse. Northern Horticulture, 48-50. (in Chinese with English abstract).

ZHOU, Y., Luo, A., 2004. Effect of organic manure on the quality of vegetables in plastic-sheet-covered sheds. Acta Agriculturae Zhejiangensis 16, 210-212. (in Chinese with English abstract).

Zhu, J.H., Li, X.L., Christie, P., Li, J.L., 2005. Environmental implications of low nitrogen use efficiency in excessively fertilized hot pepper (Capsicum frutescens L.) cropping systems. Agriculture, Ecosystems & Environment 111, 70-80.

1. * Corresponding author. *E-mail address:* acmn21@caf.ac.cn (B. Yao). [↑](#footnote-ref-1)
